# Bypassing Thermalization Losses through Ultrafast Interfacial Charge Transfer in Plasmonic Nanocavities for Water Oxidation

**DOI:** 10.1021/jacs.6c09174

**Published:** 2026-09-04

**Authors:** Yuying Gao, Christian Höhn, Jiajun Wang, Markus Wollgarten, Holger Kropf, Fengtao Fan, Can Li, Roel van de Krol, Dennis Friedrich

**Affiliations:** † Institute for Solar Fuels, Helmholtz-Zentrum Berlin für Materialien und Energie GmbH, Berlin 14109, Germany; ‡ State Key Laboratory of Catalysis, Dalian National Laboratory for Clean Energy, Dalian Institute of Chemical Physics, Chinese Academy of Sciences, Dalian 116023, China; § Institut für Chemie, Technische Universität Berlin, Berlin 10623, Germany

## Abstract

Spatially inhomogeneous plasmonic heterostructures concentrate light into nanoscale volumes and offer powerful routes for plasmon-mediated solar energy conversion. However, how hot carriers evolve and lose energy under such extreme nanophotonic confinement, particularly during interfacial transfer, remains largely unexplored. Here, we demonstrate that rationally engineered plasmonic nanocavities with intense nanoscopic field localization provide a unique platform to bypass these energy-loss channels by enabling nonthermal charge injection on a sub-40 fs time scale. Using polarization-resolved and time-resolved multiphoton photoemission spectroscopy, we reveal that the intense localized fields arising from plasmon-cavity mode coupling establish an accelerated and direct electron transfer channel across the interface. This behavior is accompanied by a distinctive inversion of the polarization dependence and by photon-energy-independent spectral features, confirming charge injection occurring prior to thermalization. Furthermore, the emergence of three-photon photoemission indicates a fundamental reconfiguration of the hot carrier generation and relaxation landscape resulting from the nanoscopic electric field within the plasmonic nanocavity. These insights establish a microscopic understanding of how interfacial charge transfer contributes to the enhanced water-oxidation activity in plasmonic nanocavity systems, offering a guiding framework for the development of advanced plasmonic photocatalysts and optoelectronic interfaces.

## Graphical abstract

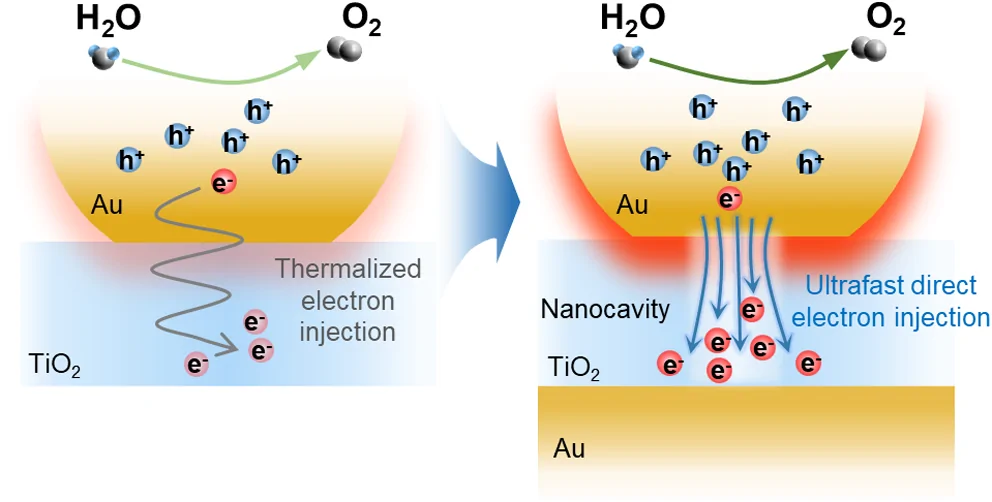

## Introduction

Plasmon-mediated charge separation at metal/semiconductor interfaces has emerged as a promising strategy for harvesting sub-bandgap photons and driving chemical transformations beyond the thermodynamic limits of conventional semiconductor photocatalysts.^1–4^ Upon surface plasmon resonance (SPR) excitation, plasmonic nanostructures generate highly non-equilibrium charge carriers, commonly referred to as hot electrons and holes, that can participate in interfacial charge transfer to drive demanding photochemical redox reactions inaccessible through band-edge excitation alone.^5–7^ Despite decades of intensive research, the quantum efficiency of plasmonic energy conversion remains fundamentally constrained by the ultrafast thermalization of hot carriers within metallic nanoparticles,^8^ which typically occurs on a few hundred femtosecond time scales and outcompetes interfacial charge separation.^9–12^ As a result, the majority of absorbed photon energy is dissipated as heat before carriers can be extracted into semiconductor components for driving chemical reactions. This intrinsic dissipation bottleneck constitutes a central challenge in plasmonic photocatalysis, motivating the development of advanced architectures capable of sustaining and directing non-equilibrium charge flow across heterointerface.

Recent advances in plasmonic nanoengineering have demonstrated that spatially inhomogeneous nanostructures can concentrate electromagnetic energy into deep-subwavelength volumes, enabling unprecedented control over light-matter interactions at the nanoscale.^13–15^ In particular, plasmonic nanocavities, comprising metallic nanostructures separated from reflective substrates by nanometer-scale dielectric spacers, provide an unique means of intensifying local electromagnetic fields through the coupling of SPR and Fabry-Pérot cavity modes.^5,16^ This intense field localization has been exploited to enhance nonlinear optical responses,^17^ surface-enhanced spectroscopies,^18,19^ and photocatalytic reactivity.^20–22^ Recent studies have further shown that plasmon-induced hot-carrier generation, relaxation, and interfacial transfer critically govern photoelectrochemical activity, underscoring the importance of resolving carrier dynamics across ultrafast temporal scales.^23–25^ These advances have established important links between photogenerated carrier behavior and catalytic performance in plasmonic systems. However, under strongly confined electromagnetic environments such as plasmonic nanocavities, the microscopic mechanisms governing interfacial charge separation remain poorly understood. In particular, whether these intense nanoscopic fields can fundamentally shift interfacial charge transfer pathways away from those in conventional heterostructures remains largely unexplored. Consequently, a significant knowledge gap persists regarding how SPR-nanocavity coupling and field orientation dictate the temporal evolution and energy distribution of hot carriers, which hinders the ability to decipher the microscopic origins of performance enhancements observed in nanostructured plasmonic photocatalysts.

To provide a complete physical picture of these dynamics, it is essential to simultaneously resolve the generation, relaxation, and transfer of hot electrons in both the temporal and energy domains. Here, we demonstrate that rationally engineered plasmonic nanocavities provide a fundamentally distinct route to interfacial charge separation. Using a model Au/TiO_2_/Au nanoparticle-on-mirror (NPoM) architecture, we combine polarization-resolved and time-resolved multiphoton photoemission spectroscopy to directly visualize hot electron dynamics and interfacial transfer with femtosecond resolution. We reveal that the intense, spatially confined optical fields generated within the nanogap drive an accelerated interfacial injection channel that outpaces intrinsic electronic thermalization. This mechanistic transition is experimentally evidenced by a distinctive inversion of polarization dependence, photo-energy-independent spectral features, and the emergence of nanocavity-induced three-photon photoemission (3PPE), providing strong spectroscopic evidence of field-enhanced non-equilibrium charge dynamics. Furthermore, we establish a microscopic foundation correlating these ultrafast carrier distributions with the substantially enhanced water oxidation performance observed in nanocavity-based photoelectrodes. By shifting interfacial charge transfer pathways from a thermally limited to a field-driven regime, this work identifies plasmonic nanocavities as a powerful platform for overcoming the fundamental efficiency bottlenecks in solar energy conversion.

## Results and Discussion

The multilayer plasmonic nanocavity combines precise structural engineering with extreme light confinement,^26^ providing a versatile platform for manipulating interfacial charge dynamics and plasmon-driven photocatalytic processes.^5,20,21^ Figure 1a schematically illustrates the studied Au/TiO_2_/Au (ATA) nanocavity architecture and its fabrication procedure through sequential deposition steps. The nanostructure consists of a 150 nm-thick continuous gold (Au) film thermally evaporated onto a cleaned indium tin oxide (ITO) substrate, serving as the plasmonic reflective mirror. A TiO_2_ dielectric spacer is subsequently deposited via atomic layer deposition (ALD) to define the nanocavity gap with atomic-level precision. Finally, discrete Au nanoparticles (NPs) are formed on the top surface, yielding a well-defined nanoparticle-on-mirror (NPoM) configuration. To better understand how this nanocavity geometry governs the plasmonic charge behavior, we also prepared a reference sample consisting of Au NPs on a TiO_2_ film without the bottom Au mirror (denoted as AT).

**Figure 1:**
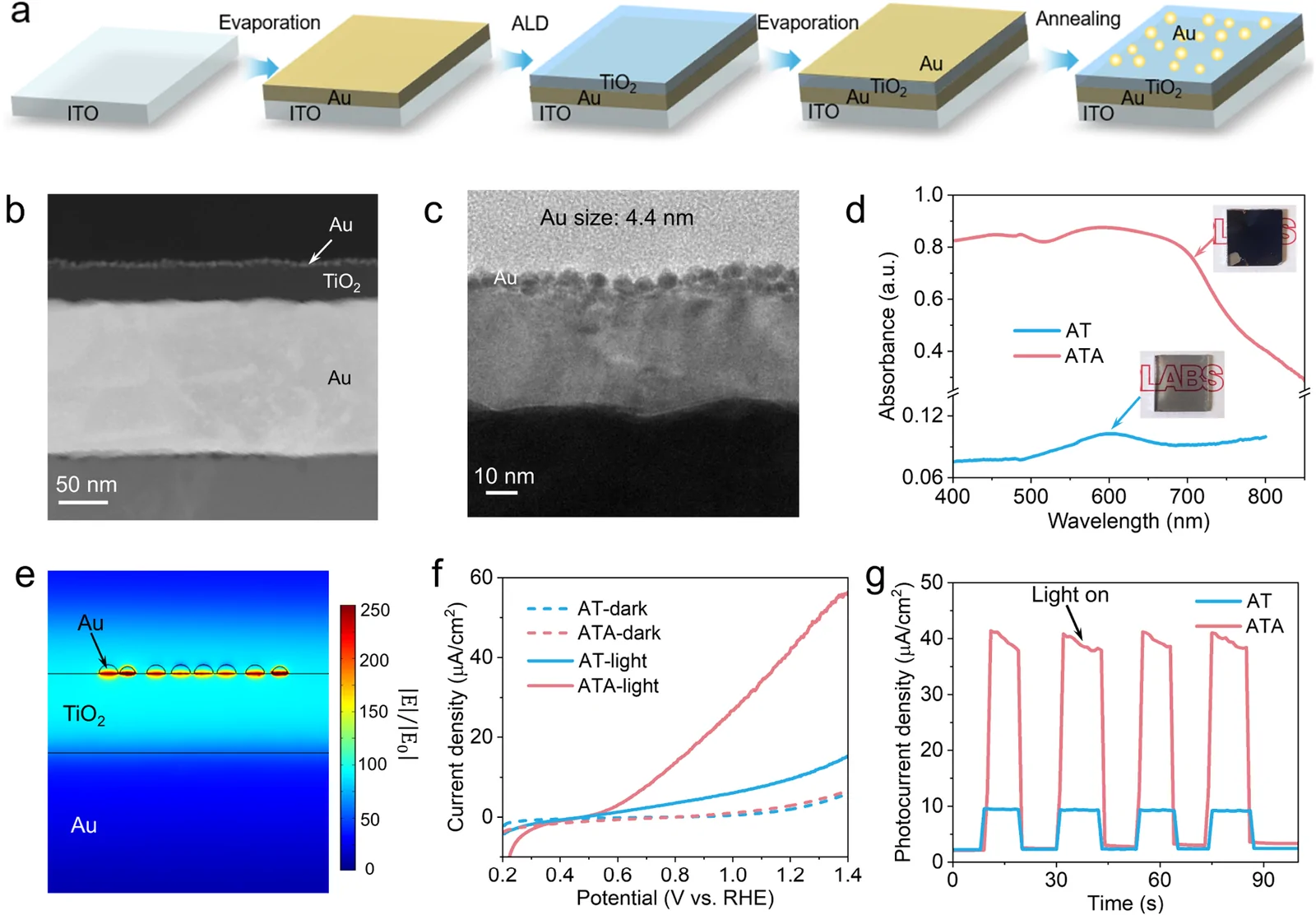
(a) Schematic illustration of the plasmonic nanocavity Au NPs/TiO_2_/Au (ATA) nanostructure fabricated via sequential deposition and thermal annealing. (b) Cross-sectional STEM image of ATA nanostructure. (c) TEM image of the ATA nanostructure, showing Au NPs with an average size of approximately 4.4 nm. (d) UV–vis absorption spectra of the ATA and AT nanostructures; inset shows photographs of the two samples. (e) Simulated electric-field distribution of the ATA nanostructure under 580 nm excitation. (f) Linear sweep voltammograms of the ATA and AT photoelectrodes measured in the dark and under visible-light illumination (λ > 420 nm). (g) Transient photocurrent responses recorded over consecutive light on/off cycles at an applied potential of 1.23 V vs. RHE for the ATA and AT photoelectrodes.

The structural features of the resulting nanocavity were characterized using cross-sectional transmission electron microscopy (TEM)/scanning transmission electron microscopy (STEM). As illustrated in Figure 1b, the bottom Au film and the top Au NPs are spatially separated by a continuous TiO_2_ spacer layer. The Au NPs are in intimate interfacial contact with the TiO_2_ surface, forming well-defined metal–semiconductor junctions that facilitate photoinduced charge transfer across the interface. The TiO_2_ spacer thickness of ∼40 nm was selected to minimize optical damping losses based on full-wave electromagnetic simulations and the optical skin depth of Au.^21,27^ TEM images further reveal that the Au NPs possess an average lateral size of ∼4.4 nm (Figures 1c and S1), enabling direct detection of photoelectron emission from both the Au NPs and the buried Au/TiO_2_ interface with minimal scattering losses owing to the ∼10 nm inelastic mean free path of photoexcited electrons under the excitation conditions discussed below.^28,29^

The absorption spectrum of the AT sample displays a single plasmonic resonance band centered at ∼600 nm (Figure 1d), which is characteristic of the SPR effect of Au NPs supported on TiO_2_ with a high refractive index.^22^ While the AT sample remains semi-transparent, the ATA architecture appears optically dark in color, indicative of a significant suppression of light transmission (Figure 1d, inset). Specifically, the ATA sample exhibits a near-perfect visible-light absorbance of ∼87% across the visible spectrum (400–750 nm), corresponding to an ∼8-fold enhancement relative to the AT control sample. This remarkable enhancement arises from destructive interference between directly reflected light and light undergoing multiple internal reflections within the TiO_2_ spacer,^21^ leading to pronounced electromagnetic field confinement within the nanocavity formed by the Au NPs and the Au mirror (Figure 1e). The resulting field amplification at the nanoparticle locations accounts for the substantially increased optical absorbance, effectively concentrating far-field radiation through coupled SPR-nanocavity modes.

Given the pronounced optical contrast, we next investigated how the nanocavity configuration modulates charge transfer dynamics and associated photocatalytic processes. The two samples were employed as photoanodes for plasmon-mediated water oxidation, which is a kinetically demanding half-reaction in artificial photosynthesis.^30^ This reaction involves the participation of multiple holes and is thus particularly sensitive to local charge density,^31,32^ making it an ideal probe reaction for how nanocavity-mediated charge transfer governs photocatalytic performance. As shown in Figure 1f,g, both photoanodes exhibit clear photoanodic currents under visible-light illumination (>420 nm). Control experiments using pure Au films or bare TiO_2_ produce negligible responses under identical conditions (Figure S2), confirming that the observed photocurrent originates from plasmon-induced water oxidation, driven by plasmonic holes following the injection of hot electrons into the counter electrode.^5^ Temperature-dependent photocurrent responses and thermal simulations consistently demonstrate that the enhanced PEC activity of the Au/TiO_2_/Au nanocavity is governed predominantly by plasmon-induced hot-carrier transfer, rather than by photothermal effects (Figure S3). Compared with AT electrode, the ATA nanocavity exhibits a markedly enhanced photocurrent density. The photocurrent responses under chopped illumination are highly reproducible and stable (Figure 1g), indicating the absence of irreversible electrochemical degradation. At an applied bias of 1.23 V vs. RHE, the ATA electrode achieves a photocurrent density of 38.9 μA cm^–2^, which is 5.4 times higher than that of the AT electrode (7.2 μA cm^–2^). Because both samples exhibit comparable surface electronic structures (Figure S4) and similar catalytic active sites, this enhancement highlights the decisive role of nanocavity-enabled plasmonic charge separation and utilization under steady-state illumination. We note that the ATA structure exhibits an approximately eightfold increase in optical absorption, whereas this enhancement results in only a fivefold increase in photocurrent. This discrepancy likely arises from several factors: (i) partial energy dissipation through nonradiative relaxation pathways, including plasmon damping and hot-carrier thermalization; (ii) the inherently energy- and momentum-selective nature of interfacial charge transfer, which allows only a fraction of photogenerated carriers to participate in efficient injection; and (iii) kinetic limitations associated with water oxidation, particularly the rate-determining O–O bond formation step, which cannot be further accelerated simply by increasing photon flux.

To gain detailed insight into the nanocavity-induced ultrafast charge separation and interfacial transfer that underlie the enhanced photocatalytic performance, we employed time-resolved two-photon photoemission (tr-2PPE) spectroscopy to directly probe the energy-resolved ultrafast dynamics of plasmon-induced hot carriers.^33,34^ This approach enables simultaneous tracking of unoccupied states populated via SPR excitation, subsequent carrier thermalization, and interfacial injection into the TiO_2_ conduction band. One-photon ultraviolet photoemission spectroscopy (UPS) was first performed to determine the surface electronic structures and energy band alignment. As shown in Figure 2a, the valence band maximum (VBM) of the AT sample lies 2.0 eV below the Fermi level (*E*_F_). Upon integrating the bottom Au mirror in the ATA architecture, the VBM exhibits only a minor shift of 0.1 eV, attributed to the limited escape depth of photoelectrons^35^ and the comparable surface electronic structure. The surface work functions of AT and ATA were estimated to be 4.90 and 5.16 eV, respectively (Figure S5). Combined with these results, the corresponding energy level alignment of ATA is illustrated schematically in Figure 2b. The resulting Schottky barrier height at the Au/TiO_2_ interface is determined to be approximately 1.3 eV, implying that plasmon-induced hot electrons exceeding this energy threshold can be efficiently injected into the TiO_2_ conduction band.

**Figure 2:**
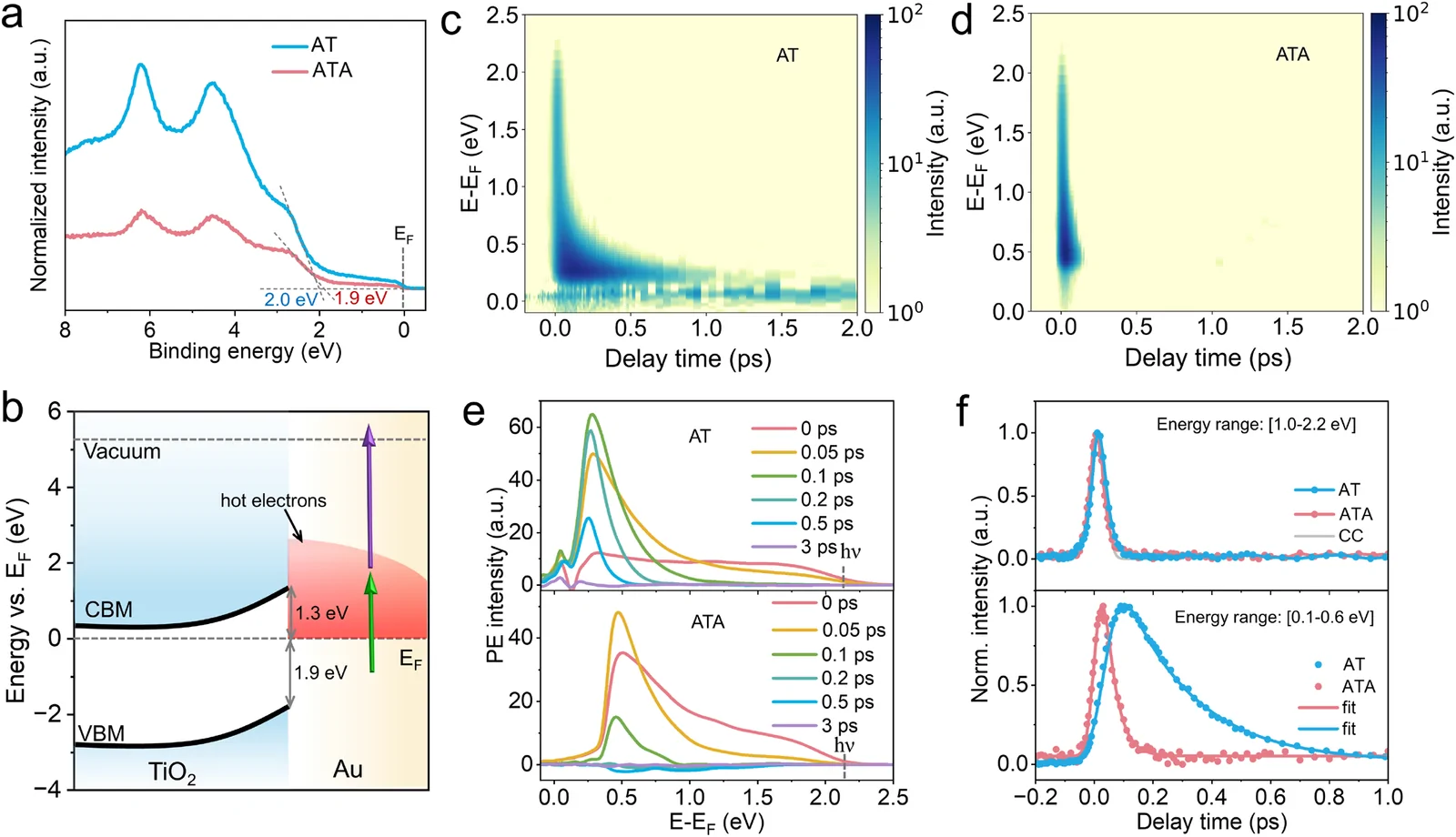
(a) Valence band spectra of the ATA and AT samples, used to determine the valence band maximum (VBM) by the extrapolation procedure. The vertical dashed line indicates the Fermi level (*E*_F_). (b) Schematic energy band diagram at the Au/TiO_2_ interface and the corresponding two-photon photoemission (2PPE) processes. CBM: conduction band minimum; VBM: valence band maximum; *E*_F_: Fermi level. (c, d) Representative two-dimensional pseudo-color 2PPE spectra for (c) AT and (d) ATA samples at a pump energy of 2.14 eV (580 nm) and a probe energy of 4.49 eV (276 nm). Background subtraction was performed using the signal recorded at −0.5 ps. (e) Photoemission (PE) intensity as a function of energy relative to *E*_F_ for AT and ATA at various pump-probe delay times. (f) Integrated dynamical traces of energy-resolved PE intensity at two spectral regions. Solid lines are fits to the kinetic model used to extract time constants, and the grey line represents the cross-correlation (CC) of the pump-probe pulses.

Figure 2c,d present the two-dimensional pseudo-colour tr-2PPE spectra for the AT and ATA nanostructures, respectively. These spectra were obtained by resonantly exciting the SPR at 580 nm (2.14 eV) and probing the transient electron distribution with a UV pulse (276 nm, 4.49 eV). The signal at −0.5 ps was subtracted as background to isolate the excited-state dynamics (raw data in Figure S6), and all energies are referenced to E_F_ to represent the transient hot-electron energy distribution. The two systems exhibit strikingly distinct spectral dynamics. In the energy domain (Figure 2e), the AT sample displays a nearly uniform distribution of electrons extending up to the pump photon energy (2.14 eV) at 0 ps delay, characteristic of the initial nonthermal carrier distribution generated by plasmon decay in metallic nanostructures.^33,36,37^ As the delay increases, this distribution transitions into an exponential decay profile, reflecting the emergence of internal thermalization via electron-electron scattering within several tens of femtoseconds. Notably, a weak and long-lived feature at ∼0.05 eV above E_F_ emerges, which can be attributed to electrons indirectly injected from Au NPs into TiO_2_ surface trap states (see details in Figure S7).

In contrast, the ATA nanocavity (Figure 2d,e) exhibits a distinctly different spectral signature. At 0 ps delay, the electron population is dominated by low-energy carriers (*E*–*E*_F_ < 1 eV), while high-energy electrons (*E*–*E*_F_ ≥ 1 eV) are markedly suppressed. The absence of a broad, pump-energy-spanning nonthermal distribution indicates that the detected carriers do not originate from conventional plasmonic hot-electron generation within Au NPs, but rather reflect a redistributed population following ultrafast interfacial extraction. The observation of a low-energy-dominated distribution at 0 ps delay implies that high-energy electrons are injected into TiO_2_ quasi-instantaneously, on a time scale limited by the laser pulse duration (∼40 fs), consistent with previously reported sub-20 fs interfacial transfer processes.^36,38–40^ These observations support a qualitatively distinct charge-separation pathway in the nanocavity, wherein intense, spatially confined fields drive interfacial charge-transfer process, enabling electrons to be directly injected into the TiO_2_ conduction band before undergoing substantial energy dissipation through electron-electron scattering in Au. This mechanism contrasts sharply with the indirect, thermally limited injection observed in AT and points to a qualitatively different charge-transfer regime from stochastic, energy-loss-dominated transport to field-driven interfacial charge separation that improves charge extraction efficiency in plasmonic photocatalysts. To quantitatively evaluate the effect of nanocavity coupling in the field-driven interfacial charge transfer, electromagnetic simulations were performed to calculate the local electric-field distribution and the corresponding hot-electron generation rate (Figure S8). The ATA nanocavity exhibits substantially enhanced field confinement, resulting in an approximately 25-fold increase in hot-electron generation compared with the AT structure. These results indicate that strong plasmon–nanocavity coupling promotes ultrafast field-driven interfacial charge injection.

The distinct interfacial electron-transfer pathways are further manifested in the carrier relaxation dynamics, as illustrated in Figure 2f, which presents the energy-resolved temporal evolution of photoexcited electrons for both samples. In the high-energy region (1.0–2.2 eV), both AT and ATA exhibit decay dynamics limited by the pump–probe cross-correlation (CC). This similarity indicates that the relaxation of high-energy electrons in both systems is faster than the instrumental response (∼40 fs), consistent with the ultrashort hot-carrier lifetimes previously reported for Ag/TiO_2_ and Ag/graphite surfaces.^34,40^ Pronounced dynamics differences emerge in the low-energy region (0.1–0.6 eV), which represents electrons with insufficient energy for interfacial injection. Quantitative fitting of the decay profiles using a Gaussian response function convoluted with an exponential decay reveals a longer lifetime for AT (0.24 ps) compared with ATA (0.038 ps). This difference is likely due to the lower charge-injection efficiency in the AT system, where indirect electron transfer leaves a greater fraction of photoexcited energy confined within the Au nanoparticle subsystem, thereby slowing the relaxation rate.^33,41^ In the ATA nanocavity, the presence of an ultrafast direct electron-transfer channel efficiently injects hot carriers from Au into TiO_2_, leading to a faster decay despite its higher absorbed energy density. This interpretation is further corroborated by the higher effective electron temperatures observed for the AT sample (Figure S9), consistent with the predictions of the two-temperature model.^42,43^

The distinct temporal behaviors of the AT and ATA nanostructures underscore the sensitivity of interfacial charge transfer to local electromagnetic field confinement. To elucidate how the excitation geometry modulates the carrier injection process, polarization-resolved 2PPE measurements were performed (Figure 3a). In these experiments, the pump polarization angle (θ) was systematically varied using a half-wave plate, while maintaining p-polarized probing and constant excitation power. Here, θ = 0° and 90° correspond to p- and s-polarization, respectively.

**Figure 3:**
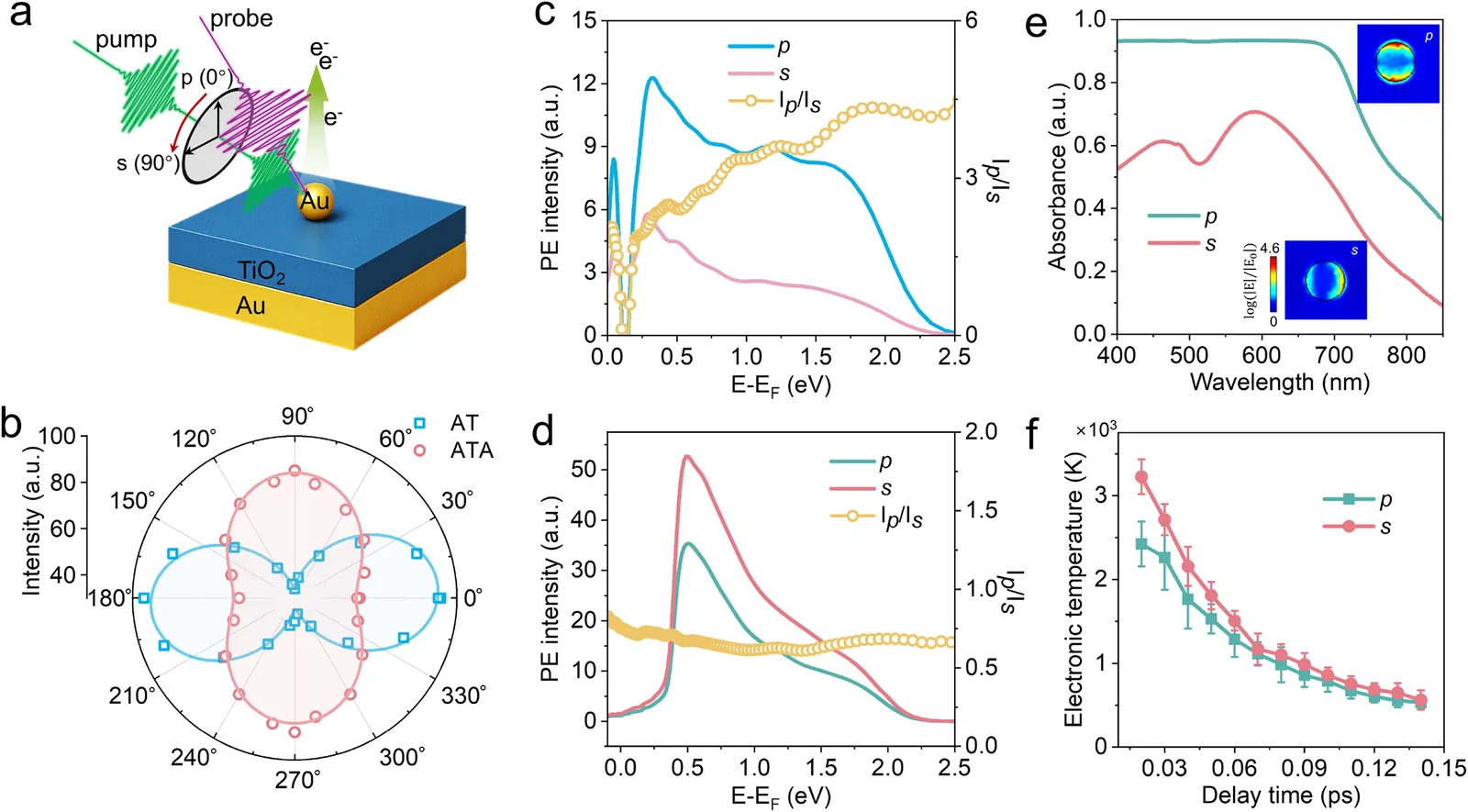
(a) Schematic illustration of the polarization-dependent tr-2PPE setup. (b) Photoemission intensity (open circles) at 0 fs delay as a function of pump polarization angle (θ) for AT and ATA. 0° and 90° correspond to *p* and *s* polarization of the pump, respectively. Blue and red lines are fits to cos^2^ θ (AT) and sin^2 ^θ (ATA) functions. (c, d) 2PPE spectra (left axis) for (c) ATA and (d) AT under *p*- and *s*-polarized pump excitation. The ratio of p- to s-polarized photoemission is shown on the right axis. (e) Absorption spectra of ATA under p- and s-polarized excitation. The inset shows simulated electric field distributions for both polarizations. (f) Time-dependent electron temperature, extracted from the high-energy tails of the 2PPE spectra by fitting Fermi–Dirac distributions under p- and s-polarized excitation.

Figure 3b displays the 2PPE intensity at an electron energy of 1.5 eV at 0 ps delay, representing the electron population capable of being injected into TiO_2_. The AT sample exhibits a conventional cos^2 ^θ dependence with maxima at *p*-polarization (0° and 180°),^44^ reflecting enhanced transition matrix elements and stronger coupling of the surface-normal electric-field component, as expected for metallic systems.^34,45^ Accordingly, the ratio of p- to s-polarized intensities (*I*_p_/*I*_s_) remains above unity and reaches values of up to ∼4.3 at higher electron energies (Figure 3c), consistent with enhanced high-energy carrier generation under *p*-polarized illumination.^33^

Strikingly, the ATA nanocavity displays an inverted polarization dependence, with maxima at *s*-polarization (90° and 270°, Figure 3b) and *I*_p_/*I*_s_ ratios below unity (∼0.6) across the entire energy window (Figure 3d). Theoretically, the 2PPE intensity scales with the local-field-induced hot electron generation and subsequent injection.^33,44^ However, both experimental absorption spectra and simulated electric field distributions (Figures 3e and S10) indicate higher energy absorption under p-polarization, this fails to explain the observed lower *I*_p_/*I*_s_ ratio, as hot electron populations are generally expected to scale with the local-field intensity.^9,31,46^

To isolate the role of interfacial charge transfer from purely electromagnetic effects, we fabricated a control plasmonic nanocavity with an insulating Al_2_O_3_ spacer (Au/Al_2_O_3_/Au) of identical geometry. This insulating barrier effectively blocks interfacial electron transfer while maintaining optical field confinement, ensuring that the measured 2PPE intensity remains exclusively linked to local-field-induced carrier generation. In this system, the *I*_p_/*I*_s_ ratio remains significantly greater than unity (Figure S11), confirming that the anomalous polarization dependence observed in the ATA configuration originates from the redistribution of hot electrons governed by interfacial transfer dynamics rather than field enhancement alone. We therefore attribute the polarization inversion in ATA to interfacial electron injection driven by the surface-normal electric-field component under *p*-polarized excitation, which selectively extracts high-energy carriers from Au, thereby depleting the high-energy carrier density within the Au NPs. The efficient scavenging of these energetic carriers under p-polarized excitation is manifested as a reduced effective electron temperature and an accelerated population decay (Figure 3f), consistent with the hallmarks of a direct electron transfer mechanism. Electromagnetic simulations reveal that p-polarized excitation generates a substantially stronger localized electric field together with an enhanced field component normal to the Au/TiO_2_ interface relative to s-polarization (Figure S12). This field redistribution favors nonthermal carrier generation and facilitates more efficient interfacial injection within the nanocavity. Despite the stronger field enhancement under p-polarized excitation, the carrier relaxation dynamics remain comparable to those under s-polarization (Figure S13). This behavior suggests a balance between increased electronic excitation and accelerated interfacial extraction, where more efficient carrier injection reduces residual electronic energy and partially compensates for slower relaxation induced by higher excitation density.

The polarization-resolved measurements establish a clear link between optical excitation geometry and interfacial electron-transfer efficiency. Building on these insights, we next investigate how the excitation photon energy influences hot-carrier population and transfer dynamics. Figure 4a presents the normalized 2PPE spectra of the ATA system recorded at 0 ps delay for varying pump photon energies while keeping the probe energy fixed at 4.49 eV (276 nm). For excitation wavelengths of 490 and 520 nm, both exceeding the energy threshold for interband transition of Au, the 2PPE spectra exhibit a narrow emission peak centered at approximately 0.46 eV above *E*_F_. The peak position remains essentially independent of the pump photon energy, which likely arises from the lower cutoff of the photoemission window imposed by vacuum-level. Notably, high-energy carrier populations are nearly absent under these conditions. This behavior is different from the observation in the AT reference configuration, which displays a pronounced low-energy distribution accompanied by extended high-energy tail (Figure S14), consistent with calculations based on Fermi’s golden rule.^37,41^ We attribute the selective enhancement of d-to-sp interband transitions in the ATA to the intense nanocavity-confined field, which preferentially amplifies transition probabilities from the high-density *d*-band, thereby outcompeting conventional intraband excitation and fundamentally reshaping the nascent carrier population at the moment of generation.

**Figure 4:**
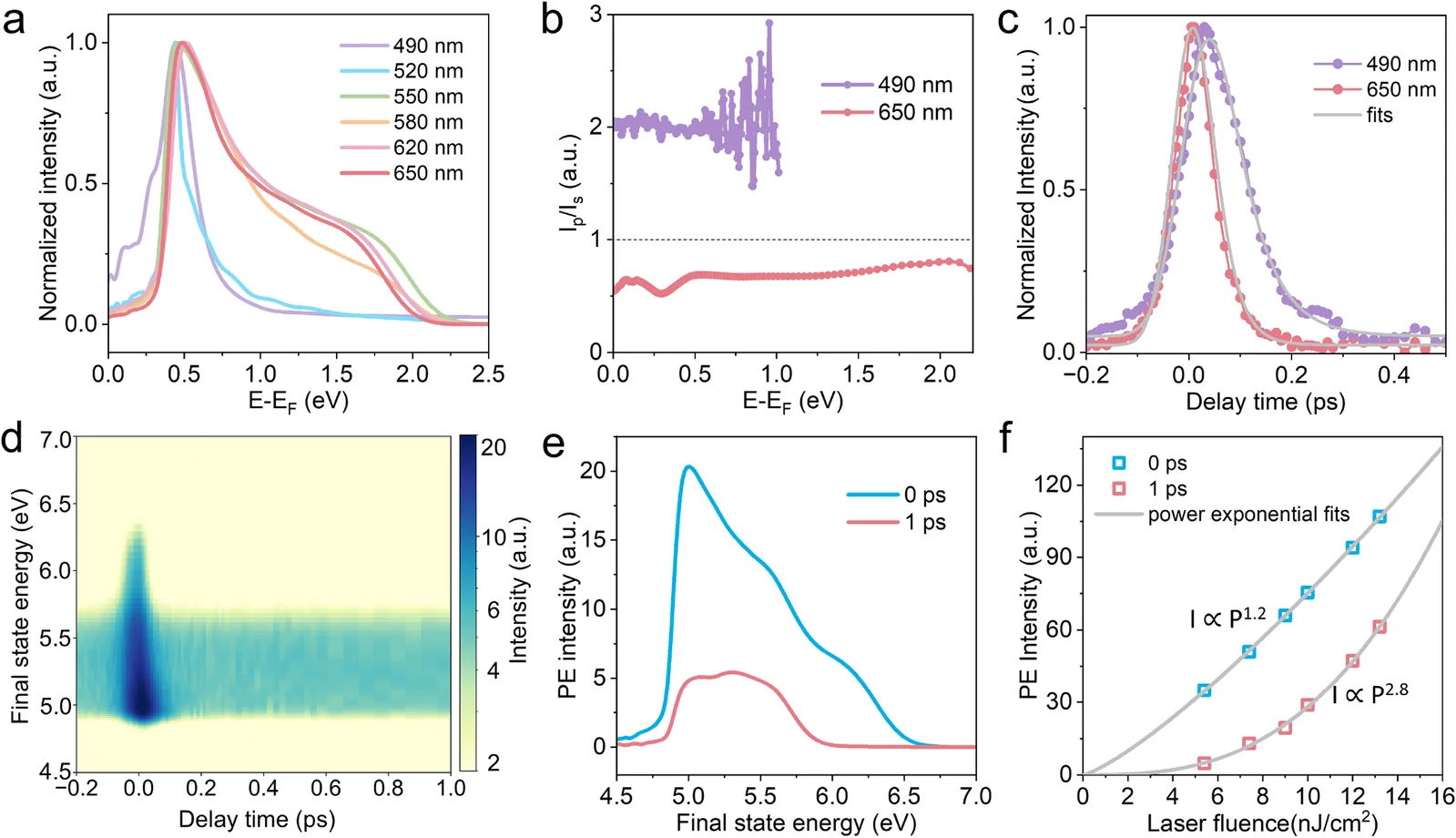
(a) Normalized 2PPE spectra of ATA at 0 fs delay under various pump energies. (b) Ratio of p- to s-polarized photoemission for ATA excited at 490 and 650 nm. (c) Normalized photoelectron intensity at 0.5 eV above *E*_F_ as a function of delay time for 490 and 650 nm excitation. (d) Two-dimensional pseudo-color 2PPE spectrum of ATA under a pump energy of 1.91 eV (650 nm). (e) 2PPE spectra of ATA at 0 and 1 ps delay times. (f) Photoemission (PE) intensity (open squares) as a function of pump fluence at 0 and 1 ps. The grey lines are power-law fits.

At excitation wavelengths longer than 520 nm, the 2PPE spectral profile remains remarkably consistent, while the upper energy edge scales linearly with the pump photon energy. Above ∼0.5 eV, the 2PPE intensity exhibits a sharp decline, consistent with the spectral characteristics observed in Figure 3. The nearly invariant 2PPE spectral shape under varying excitation photon energies constitutes a characteristic signature of direct interfacial injection pathway, indicating that charge transfer occurs prior to carrier thermalization. In this regime, the distribution and transfer efficiency of injected carriers are governed predominantly by the effective interfacial transition pathway and nanocavity-enhanced local field confinement, rather than by the excess energy of hot carriers or their subsequent thermalization dynamics.^47^

Figure 4b compares the *I*_p_/*I*_s_ ratio at zero delay for two distinct excitation regimes: 490 nm (interband-transition-dominated) and 650 nm (intraband-transition-dominated). Under 490 nm excitation, the *I*_p_/*I*_s_ ratio exceeds unity, approaching a value of ∼2. This observation is consistent with the behavior of the AT reference structure (Figure S15a,b), where *p*-polarized excitation enhances hot electron generation but yields predominantly low-energy carriers incapable of efficient injection. In contrast, at 650 nm excitation, the *I*_p_/*I*_s_ ratio drops below unity, reflecting a pronounced inversion of polarization dependence. This inversion arises from highly efficient direct interfacial injection of high-energy electrons specifically promoted by *p*-polarized excitation, in contrast to the observation in the AT where charge transfer proceeds via an indirect pathway (Figure S15c,d). These findings establish the *I*_p_/*I*_s_ ratio at zero-delay as a sensitive and robust spectroscopic fingerprint for distinguishing direct injection from indirect thermalized transfer across both interband and intraband excitation regimes.

A comparative analysis of hot-electron dynamics under these two characteristic excitation wavelengths further enables direct assessment of how interfacial charge transfer modulates relaxation landscape within Au NPs. Figure 4c presents the 2PPE dynamics at an electron energy of 0.5 eV, revealing a pronounced pump-energy-dependent decay profile. Quantitative fitting of the experimental data yields an electron lifetime of 0.059 ± 0.003 ps under 490 nm excitation, which significantly shorten to 0.031 ± 0.003 ps at 650 nm. This accelerated decay at longer wavelengths is consistent with the previously reported for Au/GaN heterostructures,^41^ where efficient interfacial charge extraction provides an additional non-radiative cooling channel, leading to suppressed transient electron temperatures and a markedly reduced electron lifetime.

Beyond pump-energy-dependent modulation of hot-electron population and interfacial transfer dynamics, we observe that both excitation wavelength and nanocavity geometry critically influence the underlying multiphoton photoemission mechanisms. Figure 4d presents the two-dimensional pseudocolor 2PPE spectrum of ATA sample under 650 nm excitation, plotted on the final state energy scale to account for multiple photoemission pathways involving photons of different energies. In addition to the transient hot-electron populations (4.8–6.4 eV) and ultrafast relaxation within 0.1 ps, a time-independent broadband photoemission feature is clearly observed between 4.8 to 5.8 eV with nearly uniform intensity (Figure 4d,e). However, the AT sample exhibits a markedly different time-independent signal characterized by a narrow and intense Gaussian-like peak (Figure S16). This peak originates from single-photon photoemission of Fermi-level electrons induced by the UV probe pulse, as evidenced by photoemission counts that scale linearly with probe fluence but remain invariant with pump fluence (Figure S17). The broadband emission in ATA thus reflects a fundamentally distinct photoemission mechanism enabled by nanocavity confinement.

To elucidate the origin of the broadband time-independent feature, we performed power-dependent 2PPE measurements. As shown in Figure 4f, the signal intensity at 0 ps exhibits a power-law exponent of 1.2, consistent with a second-order process involving the simultaneous absorption of one visible pump photon and one UV probe photon (Figure S18). In contrast, the time-independent signal at 1 ps shows a power-law exponent approaching three (∼2.8). Given the negligible contribution of the UV probe pulse to this specific feature (Figure S18a), we attribute this emission to a three-photon photoemission (3PPE) process driven exclusively by visible pump photons. This 3PPE signature is observed consistently across varying excitation wavelengths (Figure S19), confirming its intrinsic association with the nanocavity-enhanced nonlinear optical response. Importantly, when the energy scale is referenced to E_F_ to account for photon energy offsets (Figure S20), the 2PPE spectra at 0 and 1 ps exhibit nearly identical spectral profiles at high-energy edge. This correspondence implies that the 3PPE process originates from electrons following direct interfacial injection, rather than from multiphoton thermionic emission driven by thermalized hot carriers.^48^ The emergence of 3PPE reflects the extreme electromagnetic confinement established within the plasmonic nanocavity. Such field localization not only enhances the generation of high-energy nonthermal carriers but also facilitates their ultrafast interfacial extraction prior to thermalization. Given that photocatalytic water oxidation proceeds through a multihole accumulation process and is highly sensitive to carrier concentration at catalytic active sites,^31,32^ the resulting increase in carrier injection and charge separation efficiency promotes hole accumulation at the active sites and thereby accelerates water oxidation kinetics.

These findings reveal that the plasmonic nanocavity functions as a dynamic regulator, enabling direct interfacial charge transfer to bypass intrinsic electronic energy dissipation and reshape the fundamental landscape of carrier generation, relaxation, and extraction. Unlike conventional Au/TiO_2_ architectures, where sluggish indirect transfer competes unfavorably with ultrafast carrier cooling, the nanocavity-confined system establishes an accelerated charge-separation channel driven by the intense localized fields arising from strong coupling between SPR and nanocavity modes. The oxygen evolution efficiency exceeding 90% for the ATA electrode (Figure S2c) confirms that the majority of photogenerated carriers are effectively utilized for water oxidation. Notably, although the ATA nanocavity exhibits substantially enhanced optical absorption, the improved photocurrent cannot be attributed to photon harvesting alone. Instead, the enhanced local field simultaneously promotes nonthermal carrier generation, ultrafast interfacial charge injection, and efficient carrier utilization for water oxidation, collectively leading to enhanced photoelectrochemical performance. Moreover, the intense local electric field within the ATA nanocavity may also modify the interaction between water molecules and the electrode surface. Strong oriented electric fields can promote electrochemical reactions by electrostatically stabilizing reaction transition states. The potential contribution of this field-driven modulation of the reactant-surface interaction to the enhanced photocurrent warrants further investigation. These findings are consistent with recent studies highlighting the critical role of nonequilibrium hot-carrier dynamics in governing plasmonic photoelectrochemical performance. In contrast to previous reports primarily focused on carrier generation and relaxation, our results further demonstrate that nanocavity confinement can promote an ultrafast field-driven interfacial injection pathway that effectively suppresses thermalization losses.^23,24^

It is important to note that the interpretation of tr-2PPE measurements should be considered in the context of the inherent complexity of interfacial photoemission dynamics. In particular, the observed transient signals may contain contributions from local field enhancement, surface trapping states, competing relaxation pathways, and transient modifications of the surface electronic structure or work function. The introduction of the nanocavity architecture may also induce subtle modifications to the metal–semiconductor interface, including changes in the interface dipole and local band alignment. While these factors may influence the detailed spectral response, several independent observations, including the polarization-dependent inversion behavior, pump-energy-independent spectral evolution, and the distinct contrast between AT and ATA systems, collectively support the assignment of an ultrafast, nonthermal, and field-assisted interfacial charge-transfer pathway in the nanocavity structure. Although future microscopic investigations, particularly those capable of directly resolving interfacial potential dynamics and carrier phase information, will be required to fully elucidate the nonthermal charge-transfer-induced photocatalytic mechansim, the present results compellingly demonstrate that nanoscale field confinement within NPoM geometries critically governs spatial charge separation and facilitates the direct population of high-energy, non-equilibrium carrier distributions in the semiconductor. This direct injection pathway offers a decisive strategy to circumvent ultrafast thermalization losses, thereby rationalizing the markedly enhanced water oxidation activity observed. While the ultrafast measurements cannot fully reproduce the complexity of electrochemical operating conditions, they capture the initial nonequilibrium carrier generation and interfacial transfer events that define the carrier population available for subsequent catalytic conversion, thereby establishing a direct connection between early-stage charge dynamics and steady-state photocatalytic behavior. By tracing the microscopic origins of plasmon-driven charge separation to the synergistic action of nanocavity fields and interfacial electron transfer dynamics, this work provides a robust mechanistic framework for interpreting and tailoring reactivity in nanostructured photocatalytic systems.

## Conclusion

In summary, we have demonstrated that rationally engineered plasmonic nanocavities provide an effective platform for modulating interfacial charge transfer dynamics and enhancing photocatalytic performance. By combining polarization-resolved and time-resolved multiphoton photoemission spectroscopy with nanocavity engineering, we find that strong coupling between SPR and nanocavity modes enables direct, nonthermal charge injection pathways on a sub-40 fs time scale, thereby mitigating intrinsic energy dissipation within Au NPs. This behavior contrasts sharply with that of conventional metal/semiconductor interfaces, where indirect, thermalized charge transfer competes unfavorably with ultrafast carrier cooling. Moreover, we uncover a nanocavity-induced change in the multiphoton emission pathways, reflected in the emergence of three-photon photoemission driven by nanocavity-enhanced local optical fields. Together, these results show that nanophotonic confinement not only amplifies hot-carrier generation but also reshapes the carrier relaxation and extraction landscapes, enabling interfacial charge separation to proceed before substantial carrier thermalization. These findings provide a microscopic foundation for correlating non-equilibrium carrier distributions with the enhanced water oxidation performance observed in nanocavity systems, offering a definitive spectroscopic framework to understand how nanocavity-enabled charge separation can be harnessed to overcome fundamental efficiency bottlenecks in plasmon-mediated energy conversion.

## Supplementary Material



## Notes

Detailed experimental methods; numerical simulations; sample characterization; photoelectrocatalytic performance; time-resolved two-photon photoemission spectroscopy (PDF)
