# Supplementary material for: Bypassing Thermalization Losses through Ultrafast Interfacial Charge Transfer in Plasmonic Nanocavities for Water Oxidation
Source: J Am Chem Soc. 2026 Sep 4;148(36):38774–83. doi: 10.1021/jacs.6c09174 (PMC13587661; doi:10.1021/jacs.6c09174)
Supplement: Supplementary file 1 [file ja6c09174_si_001.pdf]

Supporting Information

Bypassing Thermalization Losses through Ultrafast Interfacial Charge Transfer in Plasmonic Nanocavities for Water Oxidation

Yuying Gao[†,‡,], Christian Höhn[†], Jiajun Wang[‡], Markus Wollgarten[†], Holger Kropf[†], Fengtao Fan[‡], Can Li[‡], Roel van de Krol[†,§], Dennis Friedrich[†,*]*

[†]Institute for Solar Fuels, Helmholtz-Zentrum Berlin für Materialien und Energie GmbH, Berlin 14109, Germany;

[‡]State Key Laboratory of Catalysis, Dalian National Laboratory for Clean Energy, Dalian Institute of Chemical Physics, Chinese Academy of Sciences, Dalian 116023, China;

[§]Institut für Chemie, Technische Universität Berlin, Berlin 10623, Germany;

[*]Email: yygao@dicp.ac.cn; friedrich@helmholtz-berlin.de

Experimental section:

Sample preparation:

Indium tin oxide (ITO) substrates were first sequentially cleaned by ultrasonication in acetone, ethanol, and deionized water for 10 min each, followed by nitrogen drying for 15 min. A 100 nm thick Au film was then deposited onto the cleaned ITO substrates by electron-beam evaporation at a deposition rate of 2.5 nm min^{-1}. The film thickness was calibrated using a quartz crystal microbalance (QCM). A TiO_2 layer with a thickness of 40 nm was then deposited by atomic layer deposition (ALD) in a Picosun R-200 Advanced reactor using tetrakis(dimethylamino)titanium (TDMAT) as the titanium precursor and ultrapure water as the oxygen source. During deposition, the precursor reservoir was maintained at elevated temperature to ensure sufficient vapor delivery, and the gas lines were heated to prevent condensation. The substrate temperature was held at 170 °C, with high-purity nitrogen serving as both carrier and purge gas. Each ALD cycle consisted of sequential exposure to the metal precursor and oxidant, separated by purge steps to ensure self-limiting surface reactions. Under these conditions, an amorphous TiO_2 film was obtained with a growth rate of approximately 0.60 Å per cycle. Finally, an ultrathin Au layer (~1 nm) was deposited onto the TiO_2 surface by electron-beam evaporation. The samples were then annealed in air at 500 °C for 2 h to induce dewetting of the ultrathin Au film and form Au nanoparticles on the surface. To prepare the Au NPs/TiO_2 samples, the fabrication procedure was identical except that the initial 100 nm Au layer on ITO was not deposited. Instead, a TiO_2 film was directly grown on the cleaned ITO substrates by ALD, followed by deposition of the 1 nm Au layer and subsequent thermal annealing to form Au nanoparticles. All sample preparation and transfer steps were conducted under ultra-high vacuum (UHV) conditions to minimize surface contamination and ensure reproducible interfacial properties.

Time-resolved two-photon photoemission spectroscopy (tr-2PPE)

tr-2PPE was used to probe the energy distribution and temporal evolution of photo-induced hot electrons. All measurements were performed in ultra-high vacuum (~1.3×10^{-10} mbar) at room temperature (20.6 °C). The laser system consisted of a Coherent Vitara Ti:Sa oscillator, shaped by a grating-based compressor/expander and amplified by a Coherent RegA 9050 pumped by a Verdi 12, producing 800 nm pulses with a duration of ~50 fs (FWHM), a repetition rate of 150 kHz, and a pulse energy of 10 μJ. Part of the 800 nm beam was frequency-doubled (400 nm, BBO crystal) to pump two non-collinear optical parametric amplifiers (NOPAs). One NOPA generated a visible pump (475-650 nm), and the second NOPA produced visible pulse (520-600 nm) that were frequency-doubled to generate a UV probe (265-330 nm). Pump and probe pulses were chirp-compensated with quartz prisms, achieving ~40 fs FWHM at the sample. The beams were incident on the sample at ~45° relative to the surface normal. Photoemitted electrons were collected by a time-of-flight (TOF) detector with a 7.3° acceptance angle and ~50 meV energy resolution, positioned 3-5 mm from the surface to optimize angular acceptance and minimize secondary electron contributions. Pulse densities of the pump and probe beams were individually adjustable using neutral density filters. A Cu (111) single crystal was used to determine the cross-correlation of the pump and probe pulses. The Cu crystal was cleaned by repeated cycles of Ar^+ sputtering (650 eV, 1.5×10^{-5} mbar) followed by annealing at 500 °C for 20 minutes. The measured cross-correlation was ~40 fs, determined from surface state emission via virtual intermediate states.

Ultraviolet-visible (UV-Vis) absorption spectroscopy

UV–vis absorption spectra were recorded using a PerkinElmer Lambda 950 spectrophotometer equipped with an integrating sphere. All samples were deposited on ITO substrates and positioned at the entrance port of the integrating sphere to enable simultaneous collection of transmitted, reflected, and scattered optical components. Baseline correction was performed using a $BaSO_4$ reference standard measured under identical conditions to eliminate background

contributions and instrument response. To quantify the optical absorption of the nanostructured films, we measured both total transmittance (T) and total reflectance (R) using the integrating sphere. The effective absorbance (A) was then calculated as: A = 1 − T – R, where T and R represent the total reflectance and total transmittance, respectively. The absorption enhancement factor was calculated using the integrated absorbance over the plasmonic spectral window (500–700 nm), defined as:

$$EF = \frac{\int_{500}^{700} A_{ATA}(\lambda) d\lambda}{\int_{500}^{700} A_{AT}(\lambda) d\,\lambda}$$

where $A_{ATA}(\lambda)$ and $A_{AT}(\lambda)$ denote the wavelength-dependent absorbance of the ATA and AT structures, respectively.

X-ray photoelectron spectroscopy (XPS)

X-ray photoelectron spectroscopy (XPS) analyses were carried out using a SPECS Focus 500 monochromator coupled with a Phoibos 100 electron energy analyzer. The X-ray source was an Al Kα lamp (1486.74 eV) operated at 300 W. Measurements were performed with a photoelectron take-off angle of 90°, an X-ray source–analyzer angle of 54.7°, and a pass energy of 10 eV. High-resolution core-level spectra were recorded in 0.05 eV increments, and the resulting data were processed using Shirley background subtraction.

Ultraviolet photoelectron spectroscopy (UPS)

UPS was used to determine the surface work functions and to analyze the valence band structure of the samples. Measurements were performed at room temperature on the same setup employed for XPS. A Helium I (He-I) lamp, providing photons of 21.2 eV and operated at 35 W, served as the UV source. The UV beam struck the sample at a 50° angle relative to the surface normal, while the emitted photoelectrons were collected along the normal direction. To clearly identify the secondary electron cutoff, a bias of –5.0 V was applied between the sample and the detector.

Transmission Electron Microscopy (TEM)

Transmission electron microscopy (TEM) characterization was performed using a Zeiss LIBRA 200 FE microscope operating at an accelerating voltage of 200 kV. Cross-sectional TEM specimens were prepared by focused ion beam (FIB) milling using a using a Zeiss Crossbeam 340 system. Prior to milling, a protective Pt layer (~100 nm) was deposited on the sample surface to minimize ion-beam-induced damage. The resulting electron-transparent lamellae (~80-100 nm thick) were lifted out and mounted onto Cu TEM grids for imaging and analysis.

Numerical simulations

COMSOL Multiphysics was employed to model the electromagnetic field distribution of the Au/TiO_2 and Au/TiO_2/Au heterostructures under different polarization conditions. The system consisted of a 5 nm-diameter spherical Au nanoparticle placed on a TiO_2 substrate (refractive index = 2.45) within a 3D computational domain surrounded by air. A plane wave was incident at 45° to the z-axis, with two polarization states: p-polarized light, having its electric field in the xz-plane, and s-polarized light, with the electric field along the x-axis. Perfectly matched layers (PMLs) were applied at the domain boundaries to prevent artificial reflections, and a uniform mesh of 2 nm was used throughout. Electromagnetic field distributions were calculated at the excitation wavelength of 580 nm. Based on the simulated local electric field, the Landau damping-assisted hot-electron generation/injection rate per unit area ($\rho_{injection}$) was further estimated according to:

$$\rho_{injection} = \frac{\delta\omega Q_{geometry}}{A_{surface}(\hbar\omega)^2}$$

where $A_{surface}$ is the surface area of the Au nanoparticle, ω is the angular frequency of the incident light, and $\delta\omega$ represents the linewidth of the adsorbate states. The absorbed power ($Q_{geometry}$) associated with hot-carrier generation is given by:

$$Q_{geometry} = \frac{3\nu_f \varepsilon_0}{8} \frac{\omega_p^2}{\omega^2} \int_{surface} \left| E_{normal}(\vec{r},\omega) \right|^2 dS$$

where ν_f is the Fermi velocity, ε_0 is the vacuum permittivity, ω_p is the bulk plasmon frequency of Au, and E_{normal} represents the surface-normal component of the electric field inside the Au nanostructure.

PEC measurements

The photoelectrochemical (PEC) performance of all photoanodes was evaluated in a standard three-electrode setup. A platinum (Pt) wire was used as the counter electrode, and a saturated calomel electrode (SCE) served as the reference. The working electrode was the prepared photoanode sample, which was fully immersed in 0.5 M sodium sulfate (Na_2SO_4) aqueous electrolyte with a pH of 6.8. All experiments were conducted at room temperature.

Electrochemical measurements were performed using a CHI 660E workstation. Illumination was provided by a 300 W xenon lamp (Beijing Perfectlight), with the light directed onto the front side of the photoanode to ensure uniform excitation. Linear sweep voltammetry (LSV) was conducted by sweeping the potential in the anodic direction at a scan rate of 10 mV/s.

All potentials measured versus the Ag/AgCl reference electrode were converted to the reversible hydrogen electrode (RHE) scale according to the Nernst equation:

$$E_{\mathrm{RHE}} = E_{\mathrm{Ag/AgCl}} + 0.0591 \times \mathrm{pH} + 0.196\ \mathrm{V}$$

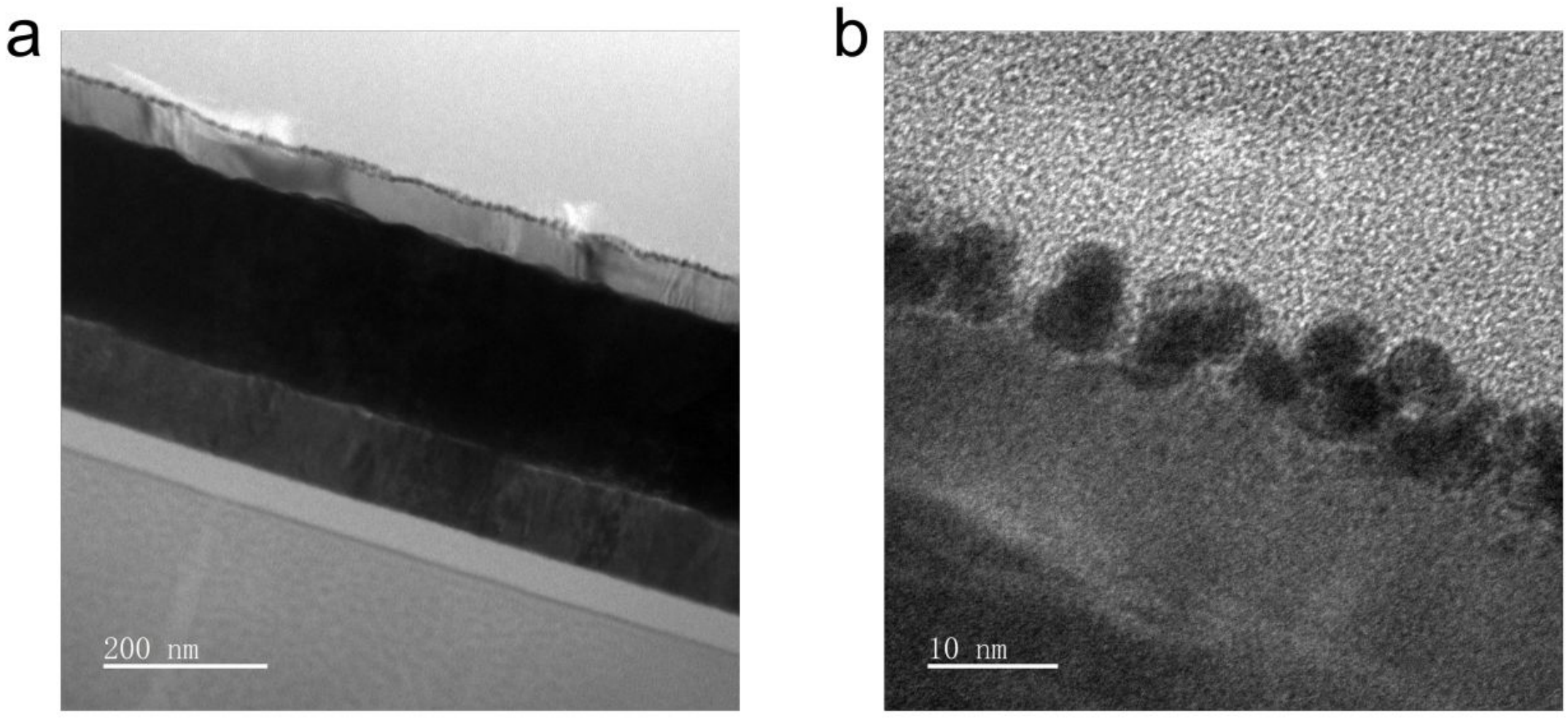

Figure S1. (a,b) Cross-sectional (a) and large-view (b) TEM images of the Au NPs/TiO_2/Au (ATA) nanostructure, revealing Au nanoparticles with an average size of 4.4 nm.

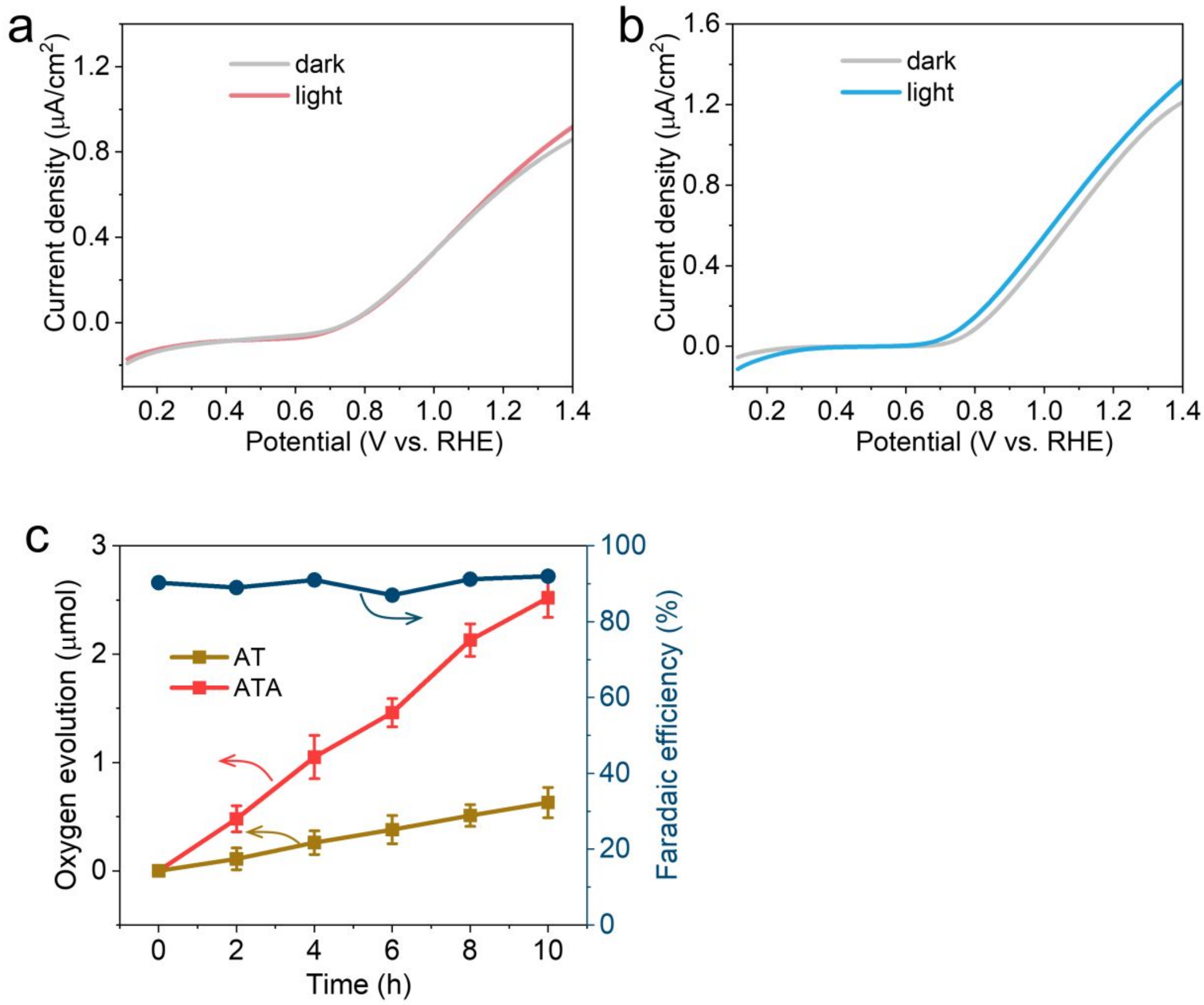

Figure S2. (a,b) Plots of current vs potential of (a) pure TiO_2 film and (b) Au film electrodes recorded in 0.5 M Na_2SO_4 aqueous solution in the dark and under visible light illumination ($\lambda >$ 420 nm). (c) Oxygen evolution versus reaction time for the AT and ATA photoelectrodes. The corresponding Faradaic efficiency of the ATA photoelectrode for photoelectrochemical oxygen evolution at 1.23 V versus RHE is plotted on the right axis.

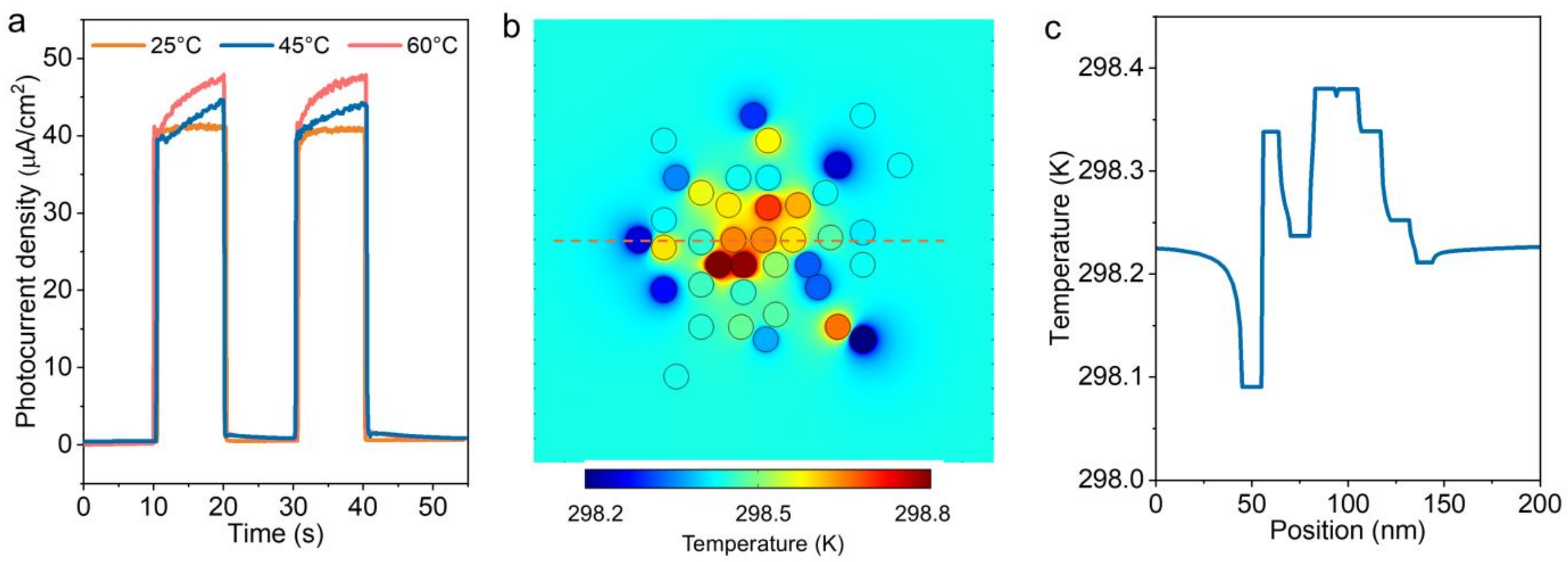

Figure S3. (a) Temperature-dependent transient photocurrent responses recorded over consecutive light on/off cycles at an applied potential of 1.23 V vs. RHE for the ATA photoelectrodes. (b) Simulated temperature field distribution for the ATA nanostructure under SPR excitation. (c) Corresponding temperature profile extracted along the white dashed line marked in (b), showing the spatial temperature gradient from the nanostructure surface into the surrounding medium.

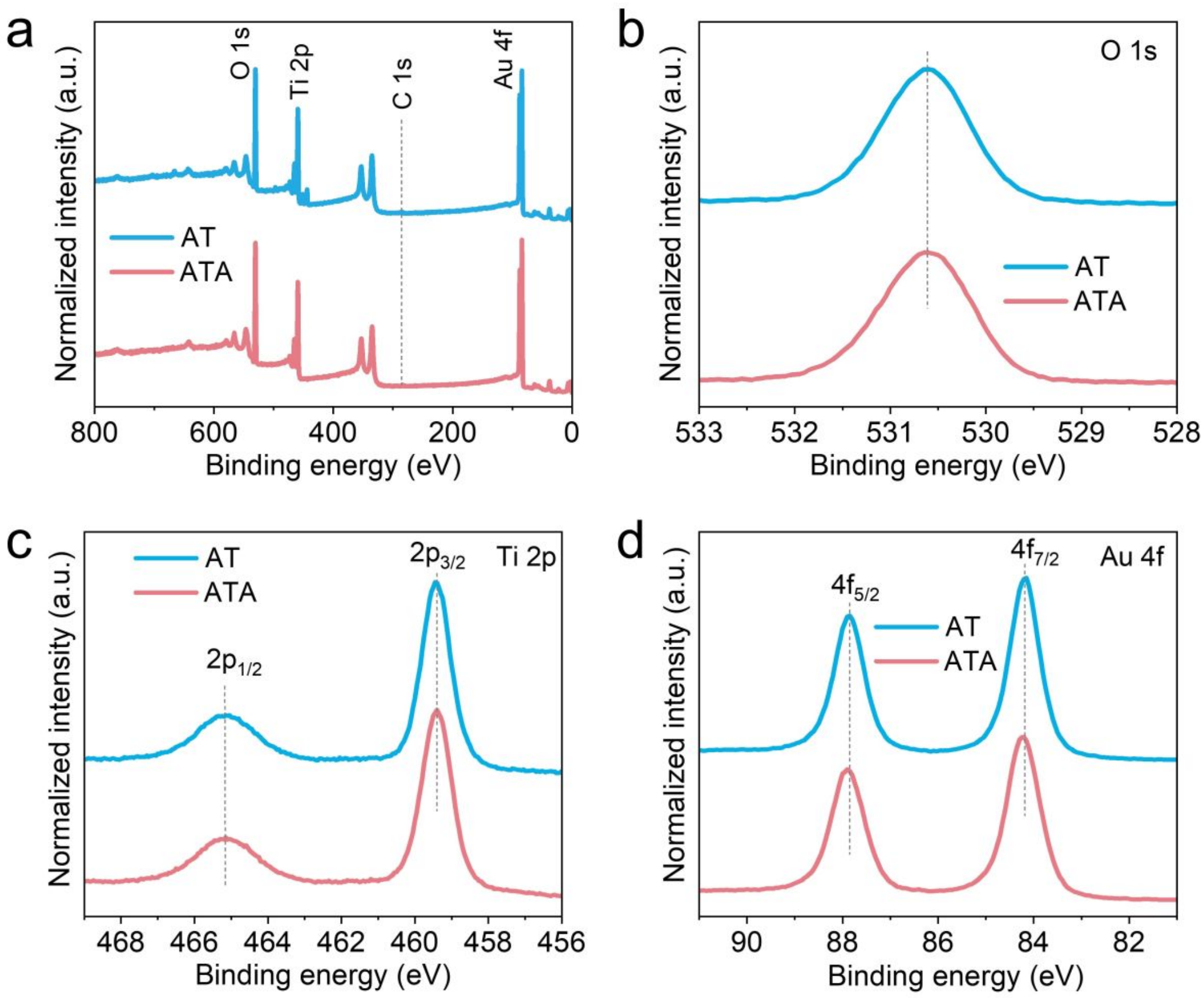

Figure S4. (a) Full XPS spectrum of AT and ATA samples. (b–d) High resolution XPS spectra of O 1*s*, Ti 2*p*, and Au 4*f*, respectively.

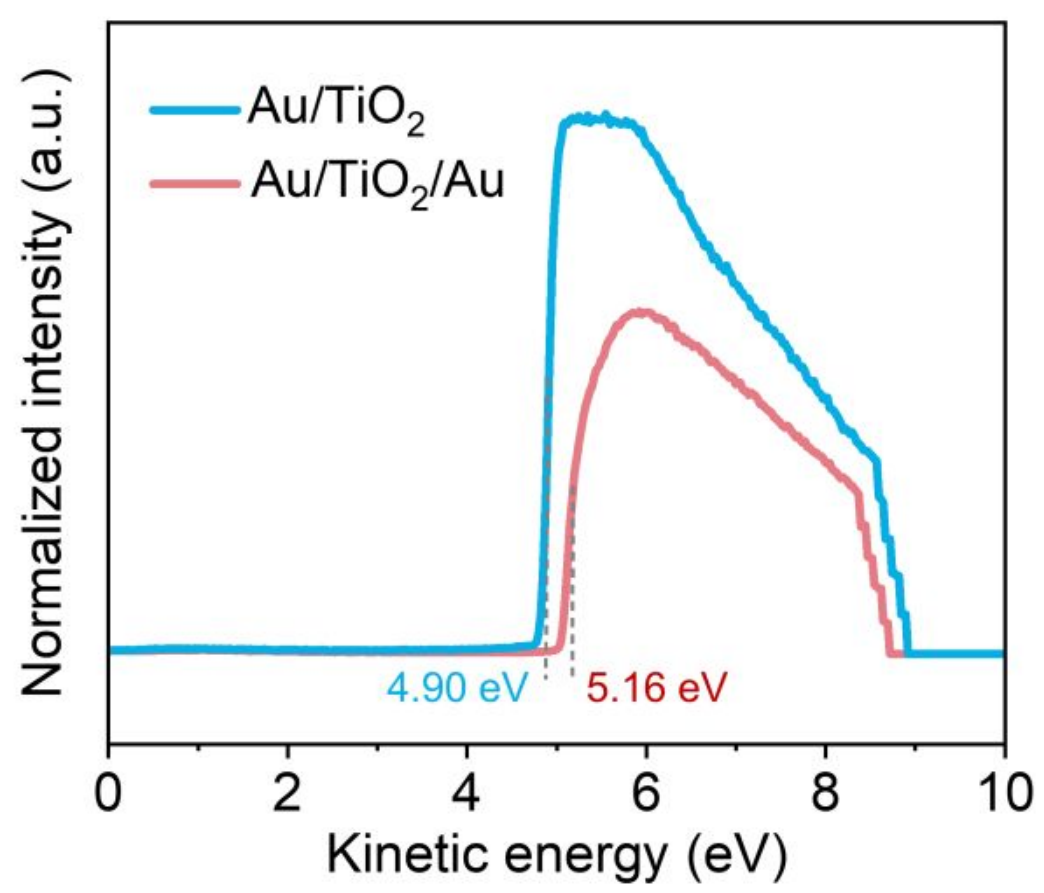

Figure S5. Work function edges in UPS for the Au/TiO_2 and $Au/TiO_2/Au$ samples.

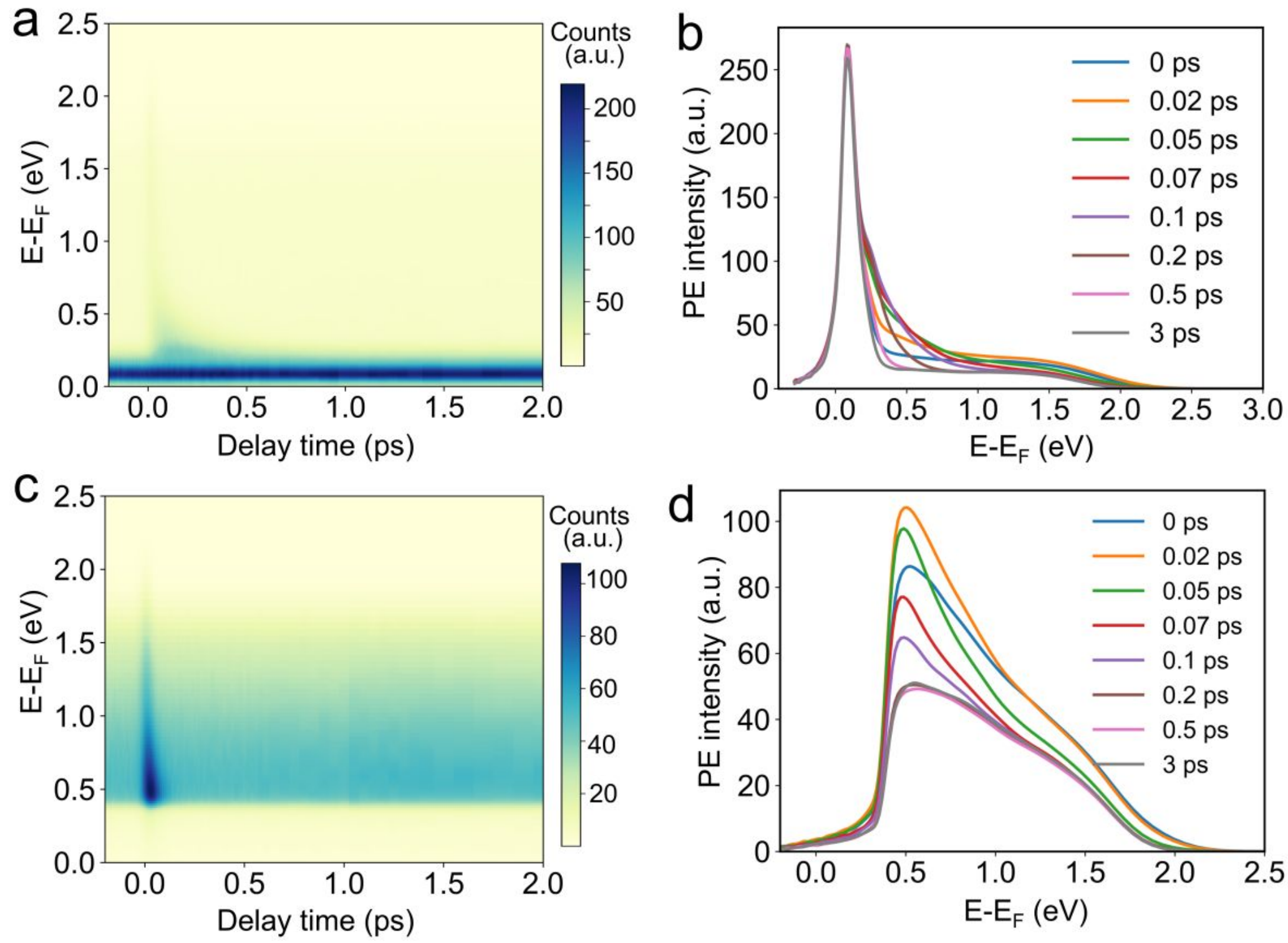

Figure S6. (a) Pseudo-color plot of 2PPE spectrum for Au/TiO_2 sample under pump energy of 2.14 eV and probe energy of 4.49 eV. (b) 2PPE spectra for Au/TiO_2 sample by varying pump-probe delay time. (c) Pseudo-color plot of 2PPE spectrum for Au/TiO_2/Au sample under pump energy of 2.14 eV and probe energy of 4.49 eV. (d) 2PPE spectra for Au/TiO_2/Au sample by varying pump-probe delay time. All measurements were conducted under p-polarized pump excitation.

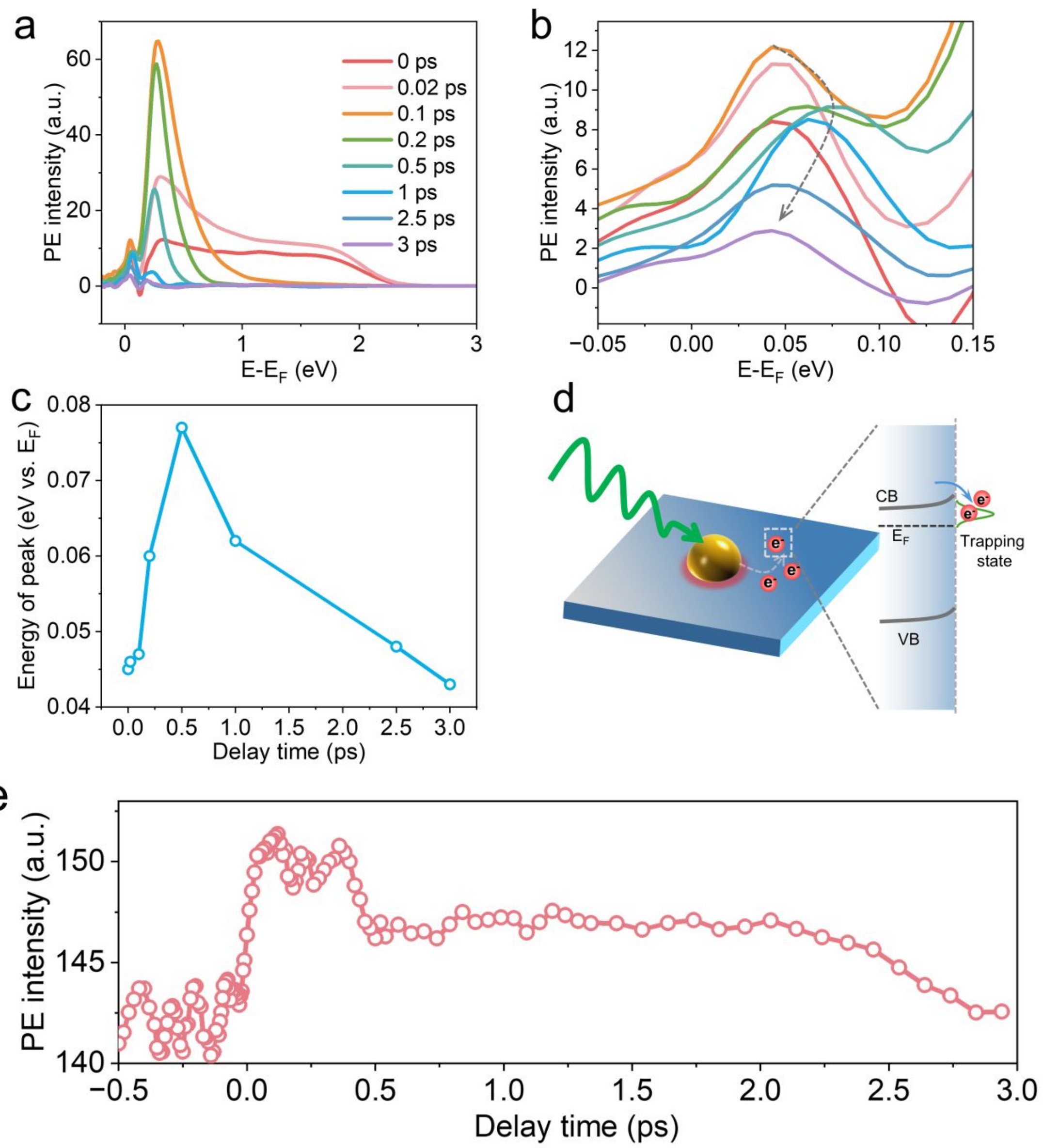

Figure S7. (a) Time-dependent 2PPE spectra of the AT sample after subtraction of the background signal acquired at a pump–probe delay of -0.5 ps. The pump photon energy is 2.14 eV and the probe photon energy is 4.49 eV. (b) Enlarged view of the 2PPE spectra in the energy range of -0.05 to 0.15 eV from panel (a), highlighting the subtle peak shift with increasing pump–probe delay time (grey dashed line). (c) Extracted peak energy as a function of pump–probe delay time. (d) Schematic illustration of plasmon-induced charge transfer and surface electron trapping at surface states near the Fermi level in the AT sample. (e) Ultrafast dynamics of photoexcited electrons at an energy of 0.05 eV. The higher signal intensity at delay times

shorter than 0.4 ps arises from the initial electron population generated upon SPR excitation. The signal intensity remains nearly constant at intermediate delay times, corresponding to electron trapping at surface states. After approximately 2 ps, a de-trapping process becomes dominant, leading to a gradual decrease in the signal intensity.

We analyzed the time-dependent 2PPE features near the Fermi level to elucidate the interfacial dynamics in the AT control sample. A distinctive, long-lived spectral feature is observed at approximately 0.05 eV above E_F (Figure S7a). Given the energy level alignment, this low-energy component with clear symmetric peak cannot be assigned to hot electrons within the Au NPs or the intrinsic TiO_2 conduction band; instead, it originates from surface trapped states within the TiO_2 bandgap. Since the pump excitation (580 nm) is below the TiO_2 bandgap, these trapped electrons likely originate from the interfacial injection of plasmon-excited carriers from the Au NPs (Figure S7c). The uniform non-thermal distribution observed at 0 ps (Figure S7a) suggests that SPR excitation first generates a non-equilibrium carrier population inside the Au NPs, followed by subsequent interfacial transfer.[1] This spectral feature confirms the predominance of an indirect charge transfer mechanism in the AT system, wherein internal thermalization within the Au NPs precedes interfacial injection. Intriguingly, the time evolution of this trap-state peak reveals a transient energy shift: the peak position increases during the first 0.5 ps before relaxing to its initial energy over a 3 ps timescale (Figure S7b). This dynamic shift is attributed to transient band flattening (or surface photovoltage effect) induced by the accumulation of injected electrons in the TiO_2.[2] The 0.5 ps rising edge of this signal is in excellent agreement with previously reported timescales for indirect injection.[3-5] Furthermore, the nearly constant intensity of the trap-state signal between 0.5 and 2.5 ps highlights the prolonged lifetime of these localized carriers (Figure S7e), distinct from the rapid decay of hot electrons in the Au NPs.

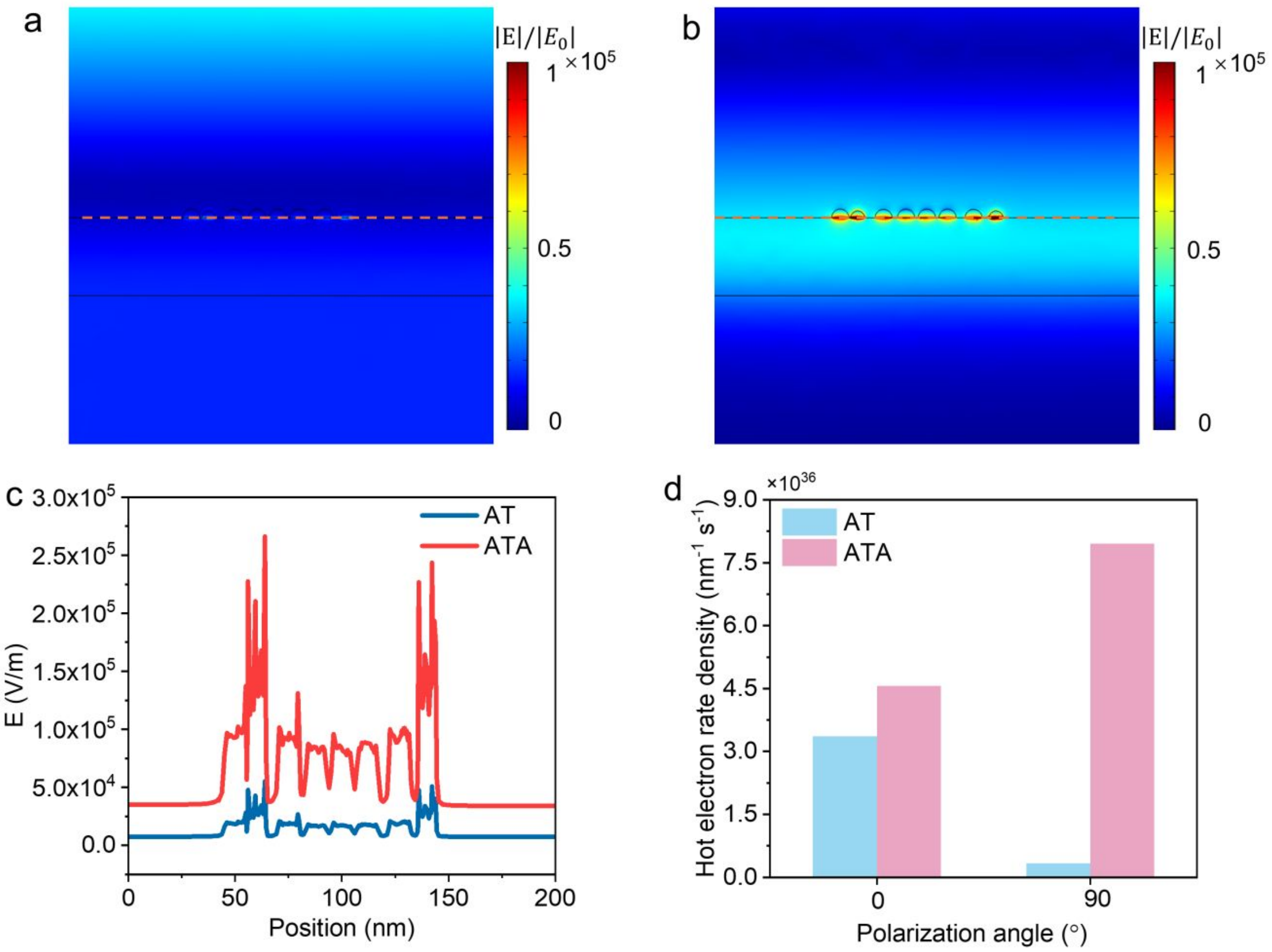

Figure S8. (a, b) Simulated electric field distributions in the XZ plane for (a) AT and (b) ATA nanostructures under p-polarized excitation. (c) Corresponding electric field profiles along the dashed lines in (a, b). (d) Hot electron generation rate density for the two samples at different polarization angles.

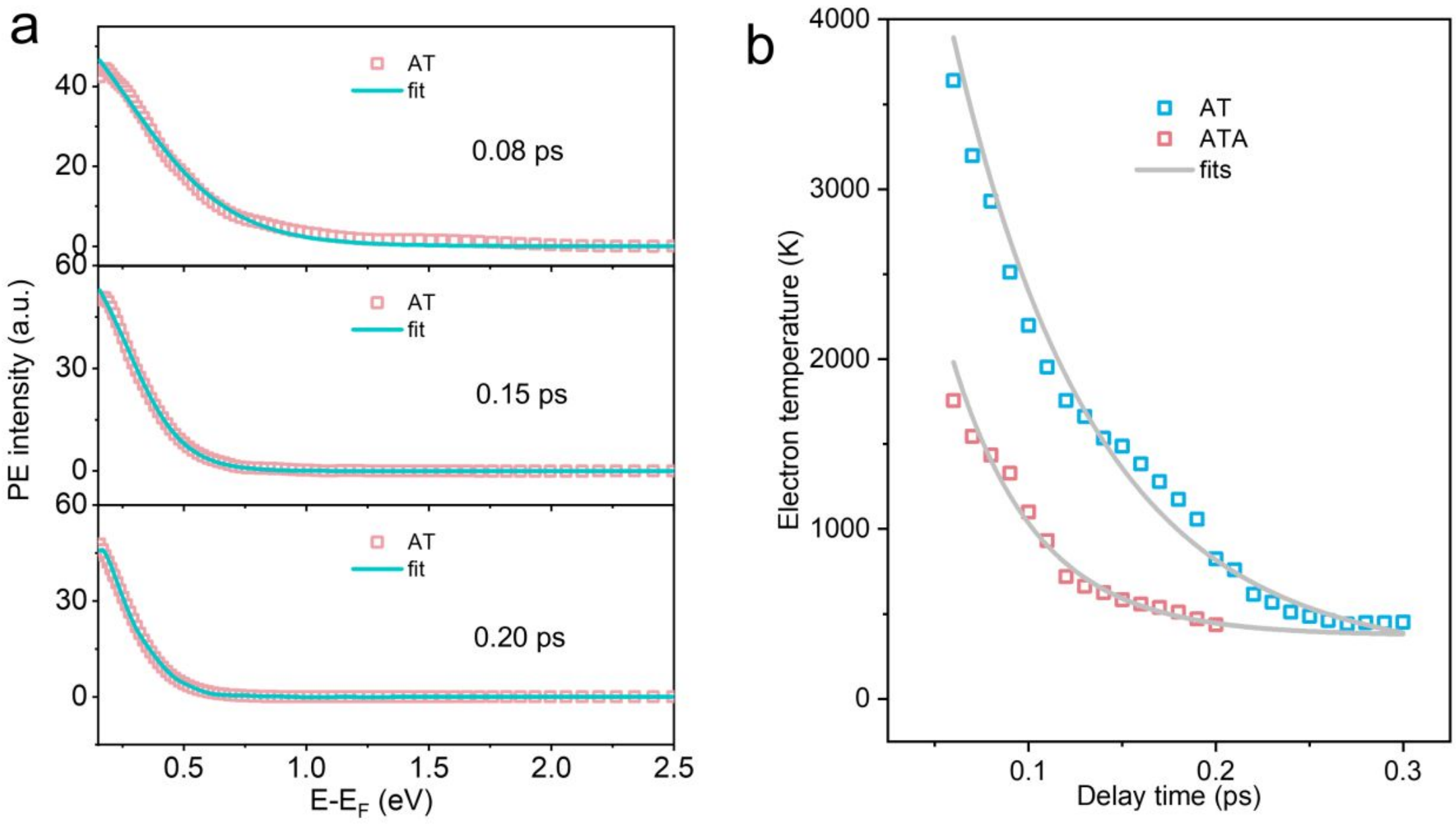

Figure S9. (a) Representative fitted high energy tails at different delay times for AT sample using the Fermi–Dirac distribution function (lines) to extract the electron temperature. (b) Extracted electron temperature as a function of time delay obtained from the fits for AT and ATA samples. The grey lines are exponential fit, yielding time constants of 0.077 ps and 0.045 ps for AT and ATA, respectively.

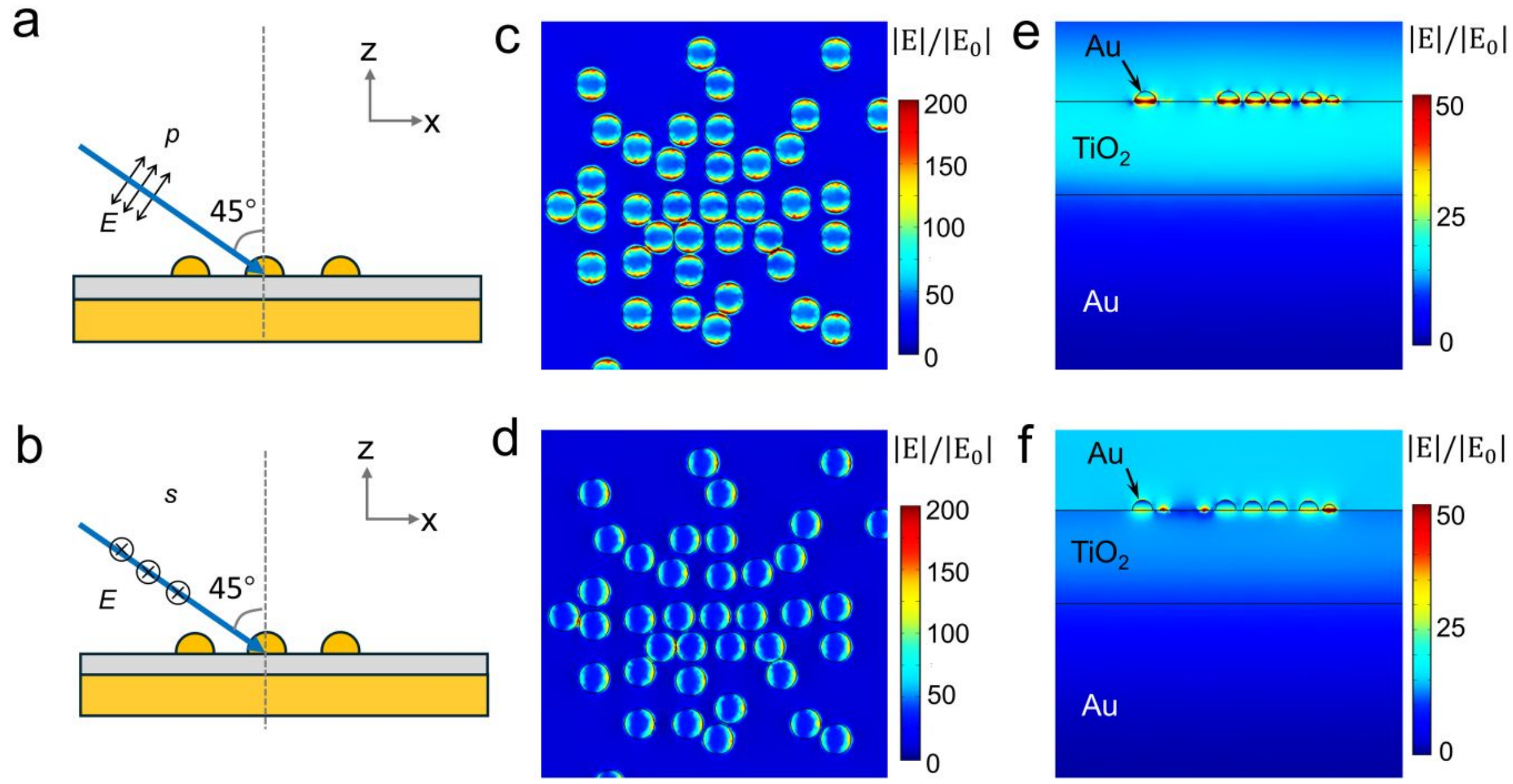

Figure S10. (a,b) Schematic representation of the ATA nanostructure for simulation model under 580 nm p-polarized excitation (a) and s-polarized excitation (b). (c,d) Simulated electric field distribution in the XY plane under p-polarized (c) and s-polarized (d) excitation. (e,f) Simulated electric field distribution in the YZ plane under p-polarized (e) and s-polarized (f) excitation.

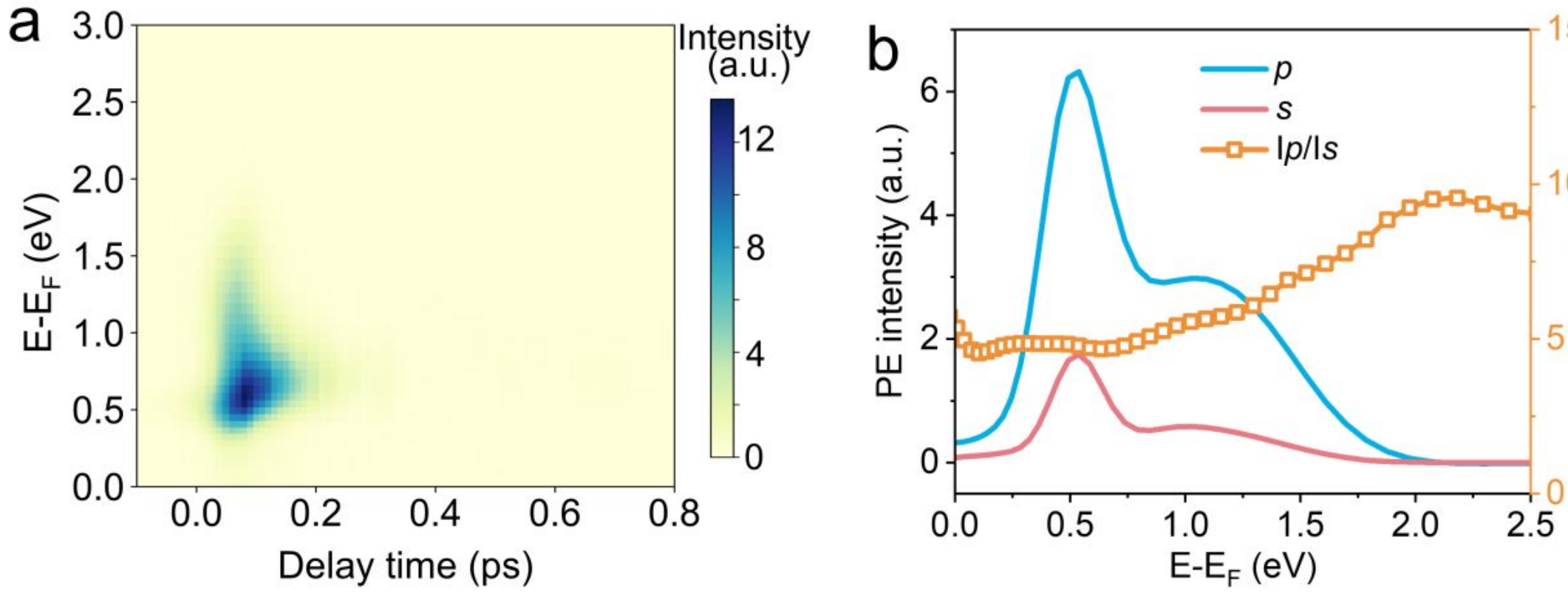

Figure 11. (a) Pseudo-color plot of 2PPE spectrum for Au/Al_2O_3/Au sample with pump energy of 2.07 eV and probe energy of 4.49 eV after background subtraction. (b) Photoelectron emission (PE) intensity and the ratio of *p*- to *s*-polarized yields as a function of excited electron energy relative to Fermi level for Au/Al_2O_3/Au under p- and s-polarized pump excitation.

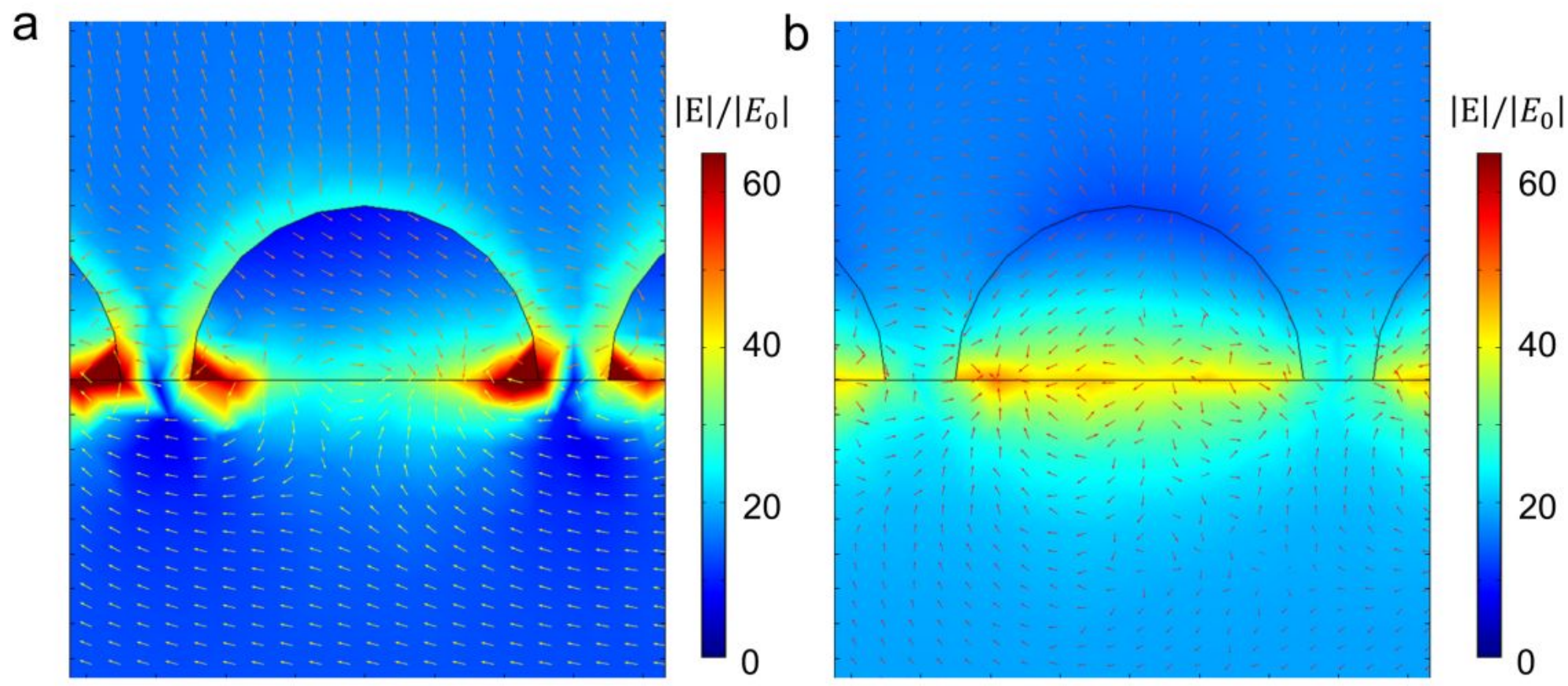

Figure S12. Simulated electric-field intensity maps and corresponding field vector distributions under (a) p- and (b) s-polarized excitation for ATA nanostructure.

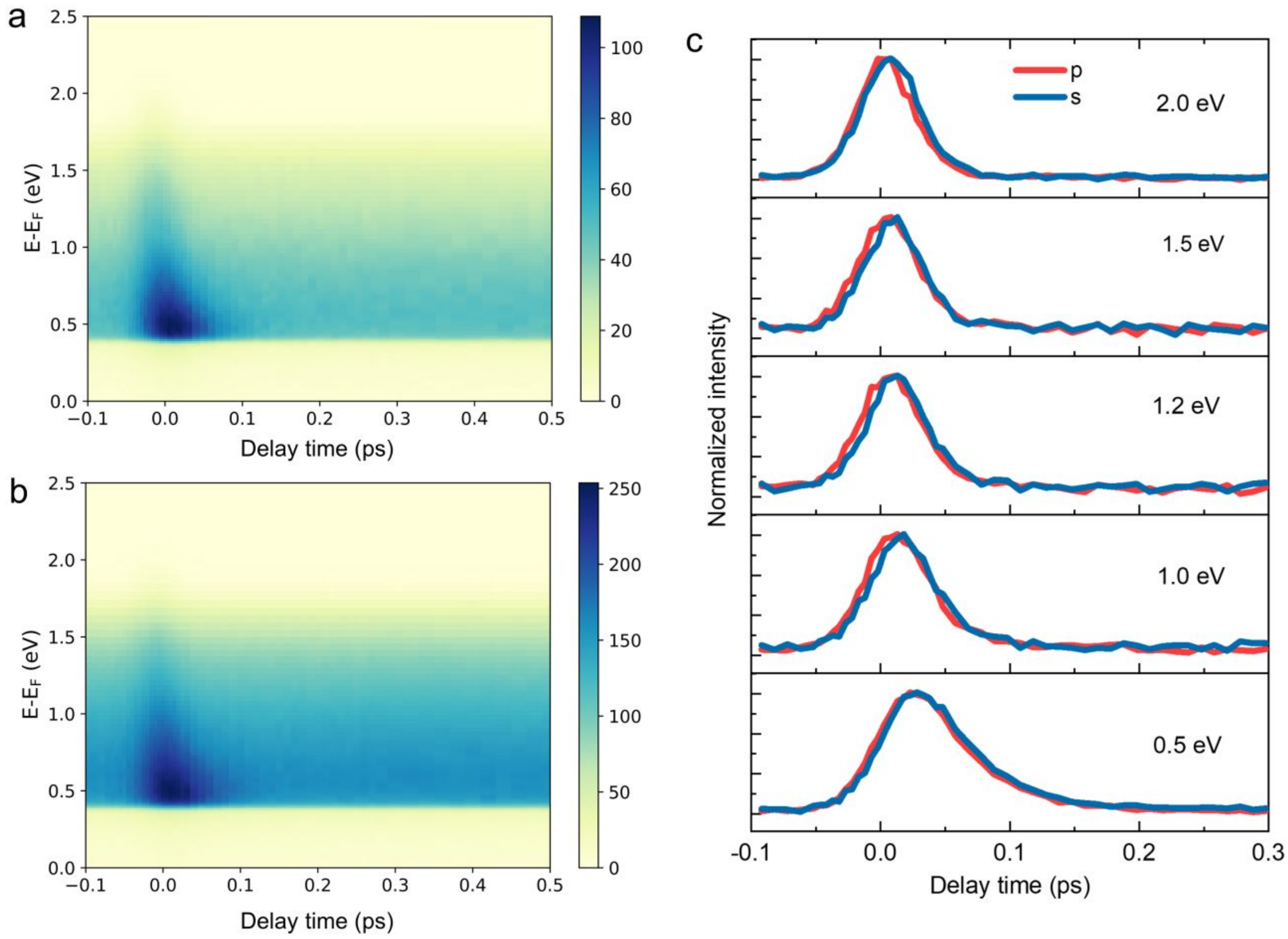

Figure S13. (a,b) Pseudo-color map of the 2PPE spectra of the ATA sample under (a) p- and (b) s-polarized excitation. (c) Energy-resolved normalized intensity as a function of pump–probe delay times.

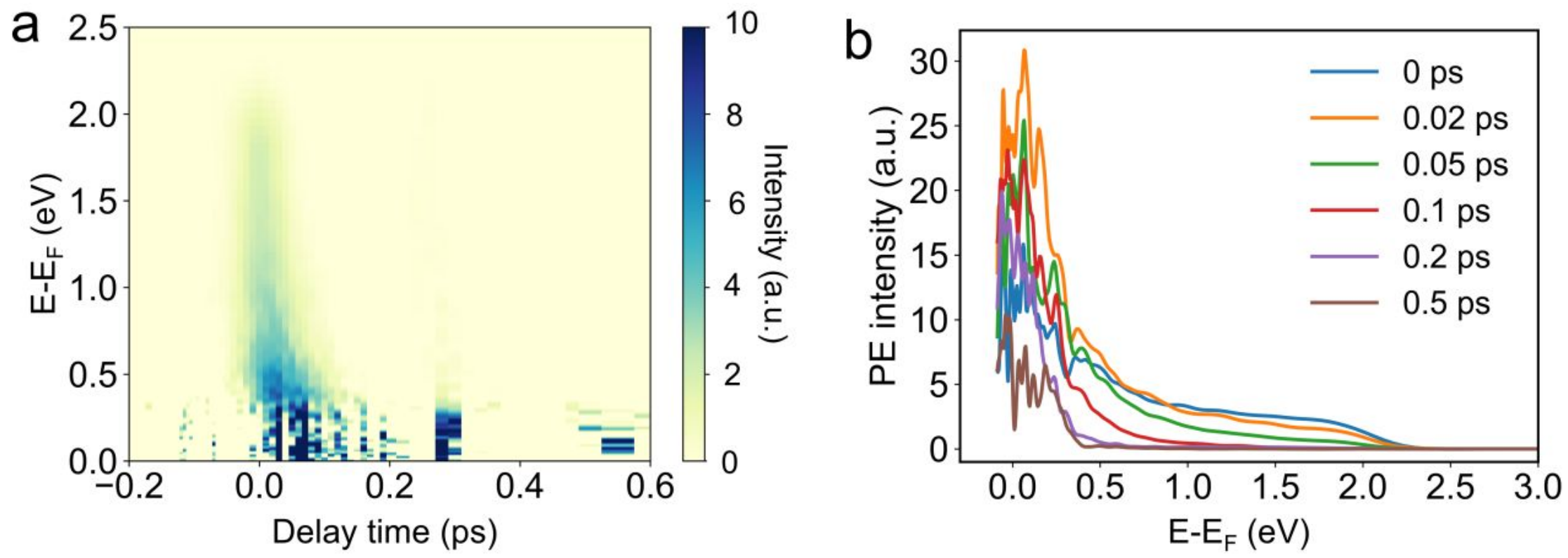

Figure S14. (a) Pseudo-color map of the 2PPE spectra of the AT sample excited with a 2.53 eV (490 nm) pump and a 4.49 eV probe after background subtraction. (b) Time-resolved 2PPE spectra of the AT sample at selected pump–probe delay times.

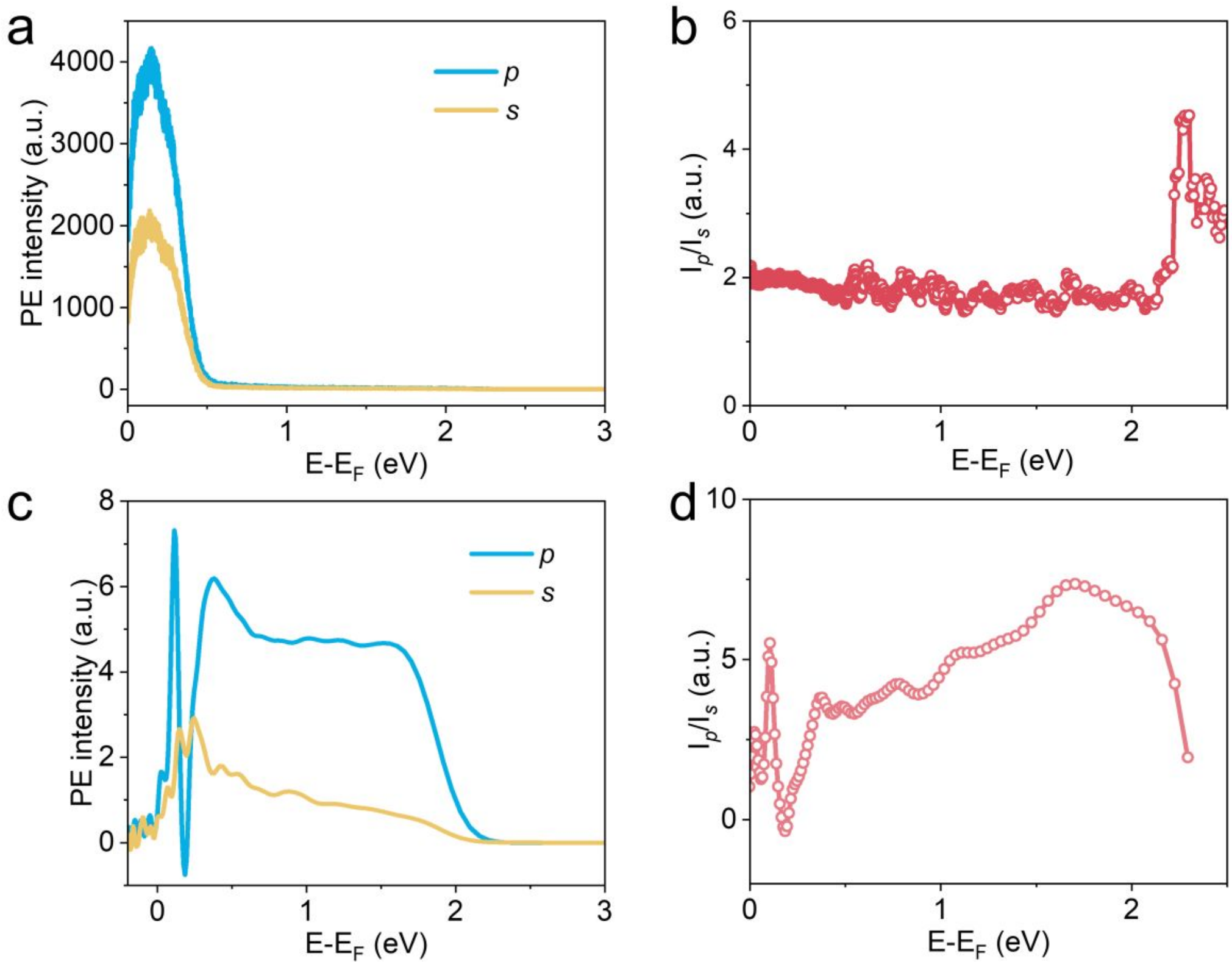

Figure S15. (a) 2PPE spectra for the AT sample under *p*- and *s*-polarized excitation. The used pump energy is 490 nm. (b) The ratio of *p*- to *s*-polarized photoemission intensity as a function of electron energy under 490 nm excitation. (c) 2PPE spectra for the AT sample under p- and s-polarized excitation with the pump energy of 650 nm. (d) The ratio of *p*- to *s*-polarized photoemission intensity as a function of electron energy under 650 nm excitation.

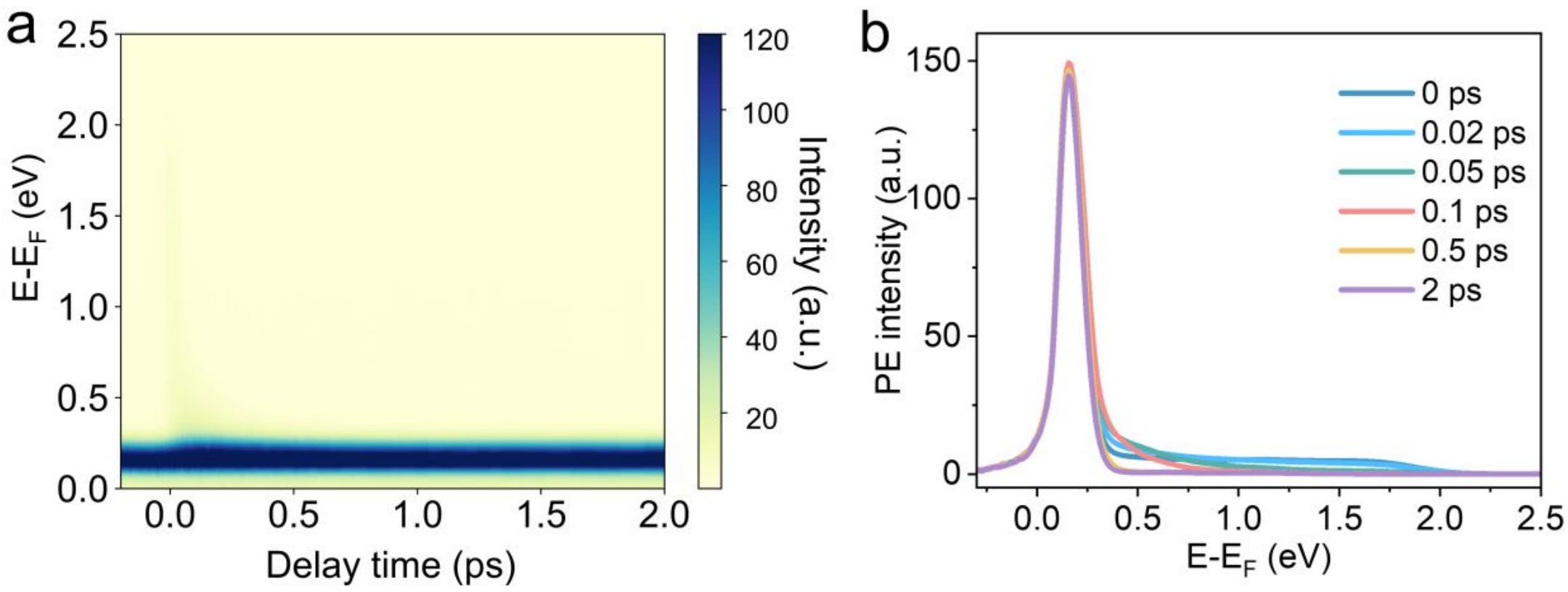

Figure S16. (a) Pseudo-color plot of 2PPE spectrum for Au/TiO_2 sample under pump energy of 1.91 eV (650 nm) and probe energy of 4.53 eV (274 nm). (b) 2PPE spectra for Au/TiO_2 sample by varying pump-probe delay time. An intense time-independent emission signal is observed at ~0.16 eV above E_F level.

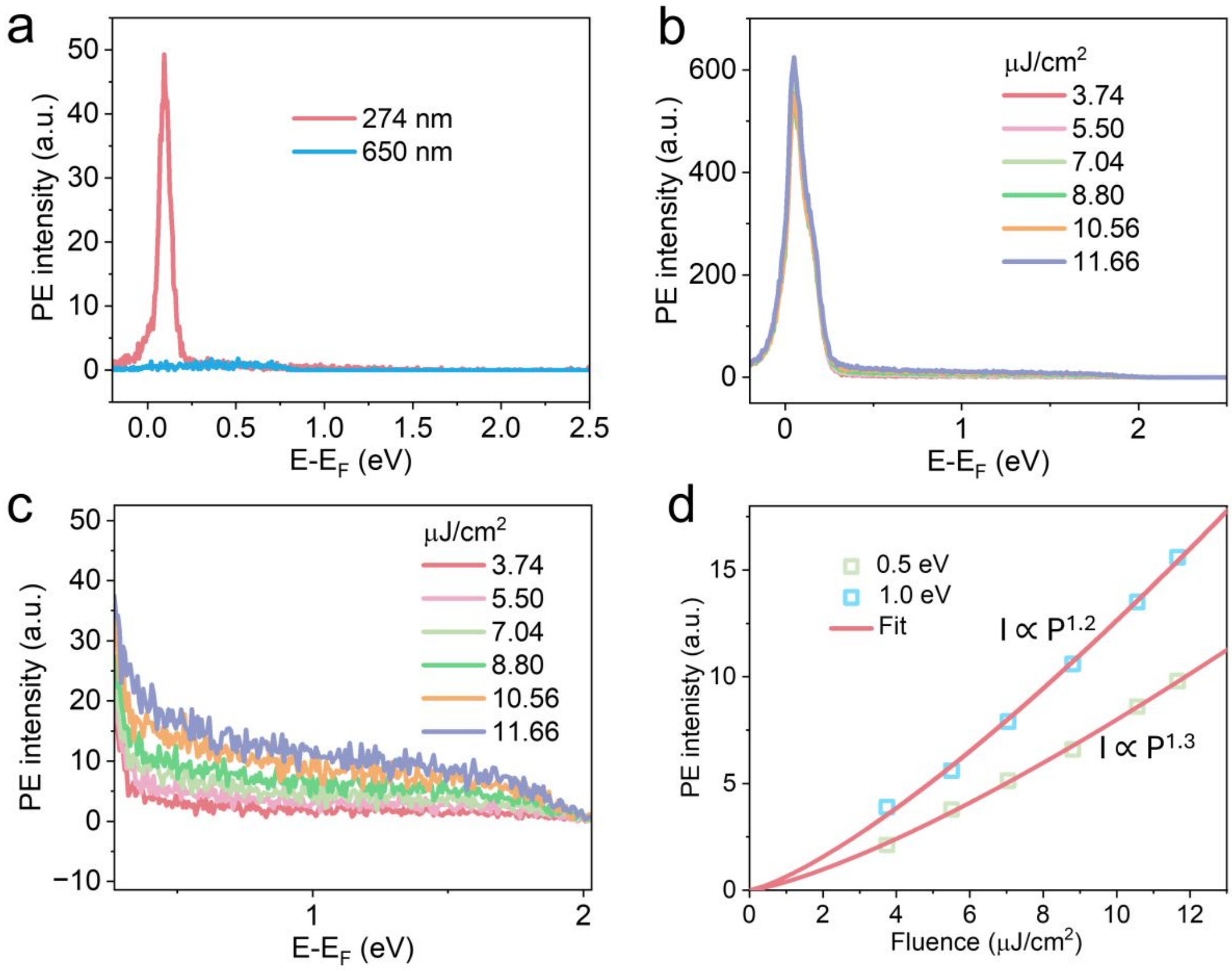

Figure S17. (a) 2PPE spectra at zero delay time for Au/TiO_2 under excitation by either a single visible pulse or UV pulses. (b) Two-photon photoelectron emission spectroscopy of Au/TiO_2 sample by varying fluences of visible pump pulse. (c) Enlarged view of 2PPE spectroscopy of Au/TiO_2 at high electron energy at different fluences of visible pump pulse. (d) Photoelectron emission (PE) intensities at electron energy of 0.5 eV and 1.5 eV at delay time of 0 fs as functions of visible pump fluences for the Au/TiO_2. The solid lines represent a power-law fit.

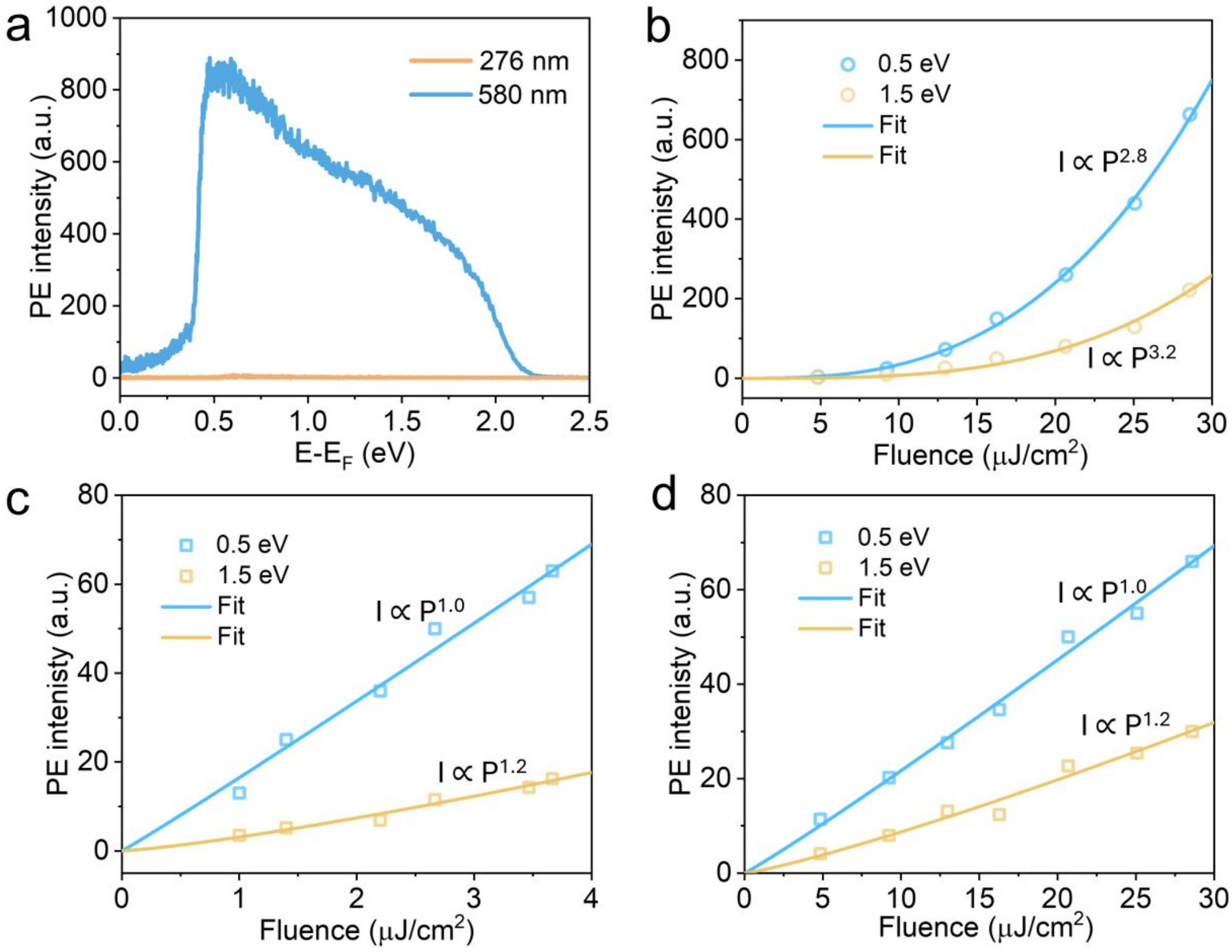

Figure S18. (a) 2PPE spectra at zero delay time for Au/TiO_2/Au under excitation by either a single visible pulse or UV pulses. (b) Time-independent photoelectron emission (PE) intensities (open squares) at 0.5 eV and 1.5 eV measured at delay time of 2 ps as functions of visible pump fluence for the Au/TiO_2/Au sample. The solid lines represent a power-law fit. (c) PE intensities (open squares) at 0.5 eV and 1.5 eV at zero delay time as functions of UV probe fluence, with pump fluence held constant, for the Au/TiO_2/Au structure. The data were background-subtracted using the signal at 2 ps. (d) PE intensities (open squares) at 0.5 eV and 1.5 eV at zero delay time as functions of visible pump fluence, with probe fluence held constant, for the Au/TiO_2/Au sample. Background signals at 2 ps were subtracted from all data.

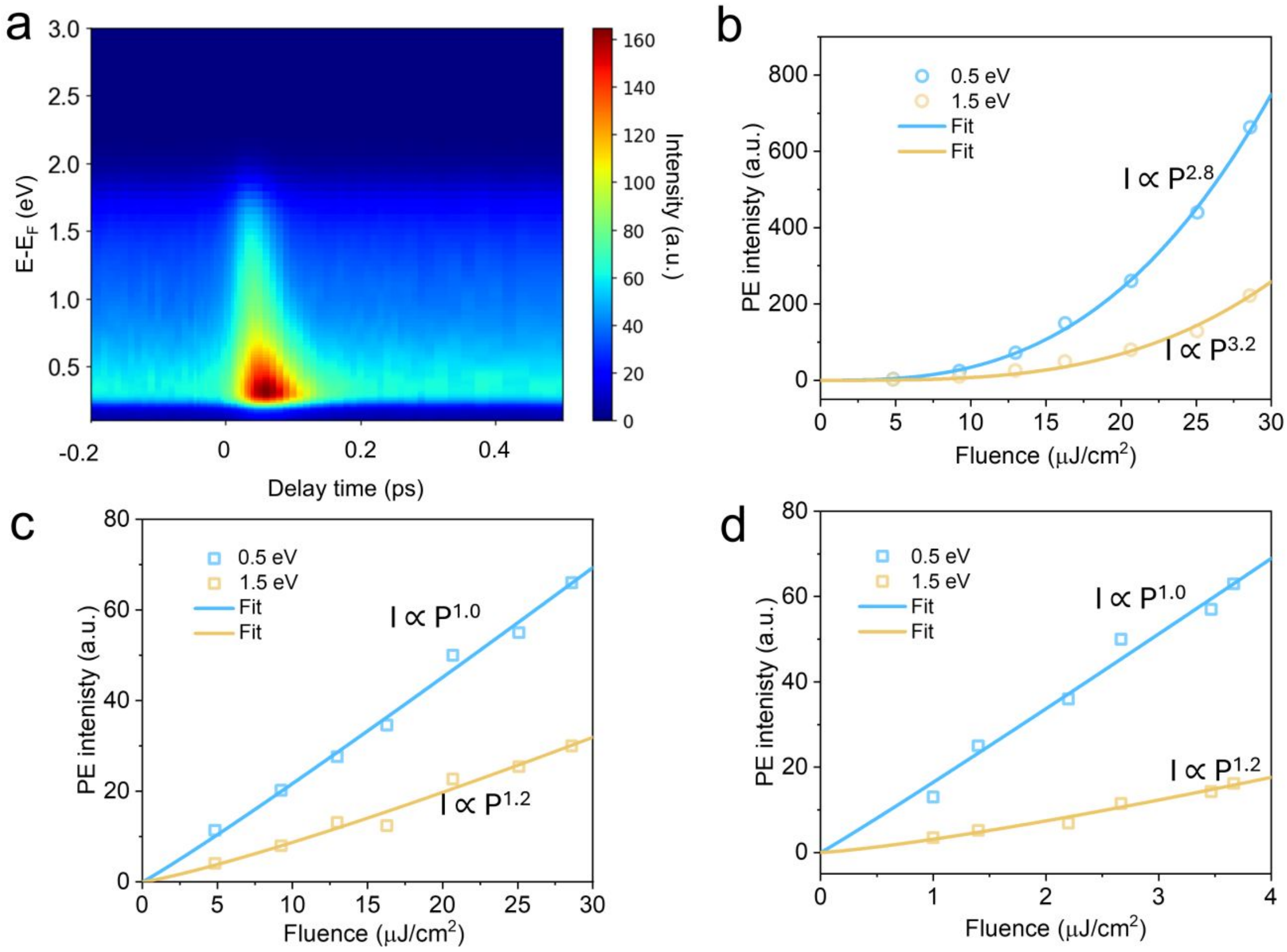

Figure S19. (a) Pseudo-color plot of 2PPE spectrum for ATA sample under pump energy of 2.25 eV (550 nm) and probe energy of 4.49 eV (276 nm). (b) Time-independent photoelectron emission (PE) intensities (open circles) at 0.5 eV and 1.5 eV measured at delay time of 2 ps as functions of visible pump fluence for the ATA sample. The solid lines represent a power-law fit. (c) PE intensities (open squares) at 0.5 eV and 1.5 eV at zero delay time as functions of visible pump fluence, with probe fluence held constant, for the ATA sample. (d) PE intensities (open squares) at 0.5 eV and 1.5 eV at zero delay time as functions of UV probe fluence, with pump fluence held constant, for the ATA nanostructure. The data were background-subtracted using the signal at 2 ps.

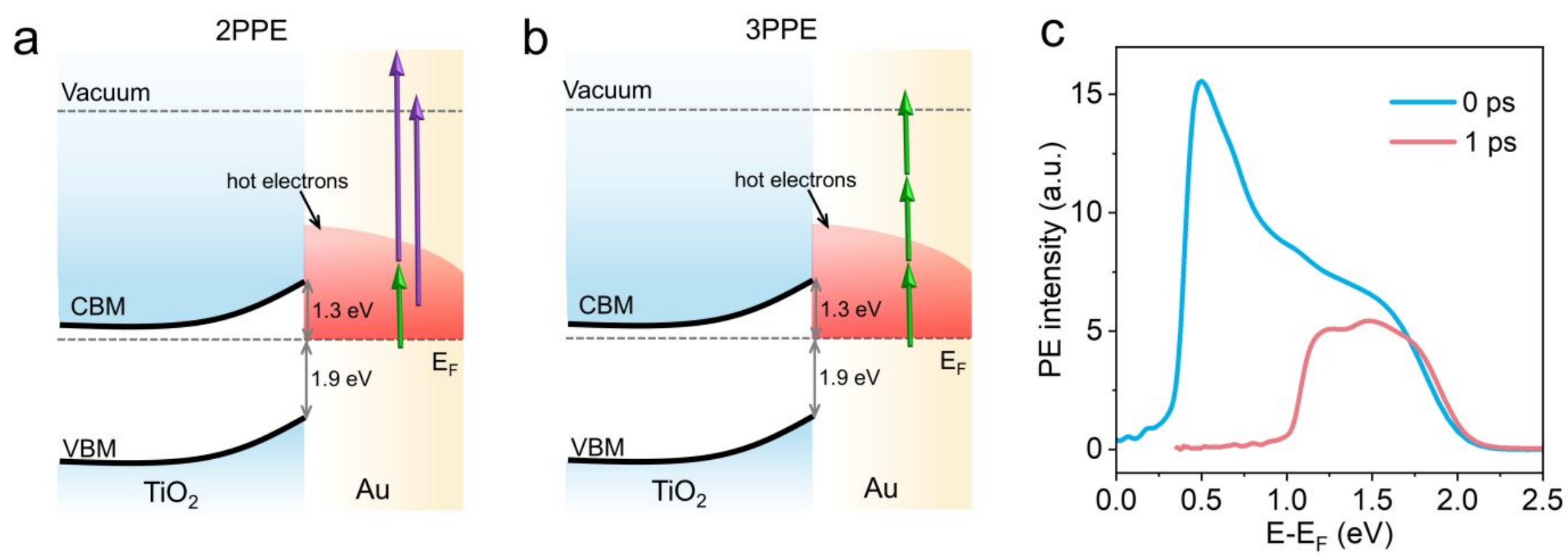

Figure S20. (a) Schematic illustration of plasmonic hot-electron two-photon photoemission induced by sequential absorption of one pump photon and one probe photon in the ATA nanostructure. (b) Schematic illustration of plasmonic hot-electron three-photon photoemission induced by the absorption of three pump photons in the ATA nanostructure. (c) 2PPE spectra of the ATA sample plotted as energy relative to the Fermi level at 0 ps delay after background subtraction and at 1 ps delay, obtained with a pump photon energy of 1.91 eV (650 nm).

Reference:

(1) Cushing, S. K.; Chen, C. J.; Dong, C. L.; Kong, X. T.; Govorov, A. O.; Liu, R. S.; Wu, N. Q. Tunable Nonthermal Distribution of Hot Electrons in a Semiconductor Injected from a Plasmonic Gold Nanostructure. *ACS Nano* **2018**, *12* (7), 7117-7126.
(2) Gierster, L.; Vempati, S.; Stähler, J. Ultrafast generation and decay of a surface metal. *Nat. Commun.* **2021**, *12* (1), 978.
(3) Ostovar, B.; Lee, S. A.; Mehmood, A.; Farrell, K.; Searles, E. K.; Bourgeois, B.; Chiang, W. Y.; Misiura, A.; Gross, N.; Al-Zubeidi, A.; et al. The role of the plasmon in interfacial charge transfer. *Sci. Adv.* **2024**, *10* (27), eadp3353.
(4) Christopher, P.; Moskovits, M. Hot Charge Carrier Transmission from Plasmonic Nanostructures. *Annu. Rev. Phys. Chem.* **2017**, *68*, 379-398.
(5) Govorov, A. O.; Zhang, H.; Gun'ko, Y. K. Theory of Photoinjection of Hot Plasmonic Carriers from Metal Nanostructures into Semiconductors and Surface Molecules. *J. Phys. Chem. C* **2013**, *117* (32), 16616-16631.
